# Recent advancements of metalenses for functional imaging

**DOI:** 10.1186/s40580-023-00372-8

**Published:** 2023-05-24

**Authors:** Dongmin Jeon, Kilsoo Shin, Seong-Won Moon, Junsuk Rho

**Affiliations:** 1 Department of Mechanical Engineering, Pohang University of Science and Technology (POSTECH), Pohang, 37673 Republic of Korea; 2 Department of Chemical Engineering, Pohang University of Science and Technology (POSTECH), Pohang, 37673 Republic of Korea; 3 POSCO-POSTECH-RIST Convergence Research Center for Flat Optics and Metaphotonics, Pohang, 37673 Republic of Korea

**Keywords:** Metasurface, Metalens, Phase modulation, Inverse design, Tunability, Numerical aperture, Aberration correction, Imaging system, Spectrometer

## Abstract

Metasurfaces can modulate light with periodically arranged subwavelength scatterers, and they can generate arbitrary wavefronts. Therefore, they can be used to realize various optical components. In particular, metasurfaces can be used to realize lenses, so-called metalenses. In the last decade, metalenses have been actively studied and developed. In this review, we firstly introduce the fundamental principles of metalenses in terms of materials, phase modulation method, and design method. Based on these principles, the functionalities and the applications can consequently be realized. Metalenses have a much larger number of degrees of freedom compared with that of existing refractive or diffractive lenses. Thus, they afford functionalities such as tunability, high numerical aperture, and aberration correction. Metalenses with these functionalities can be applied in various optical systems such as imaging systems and spectrometers. Finally, we discuss the future applications of metalenses.

**Graphical Abstract:**

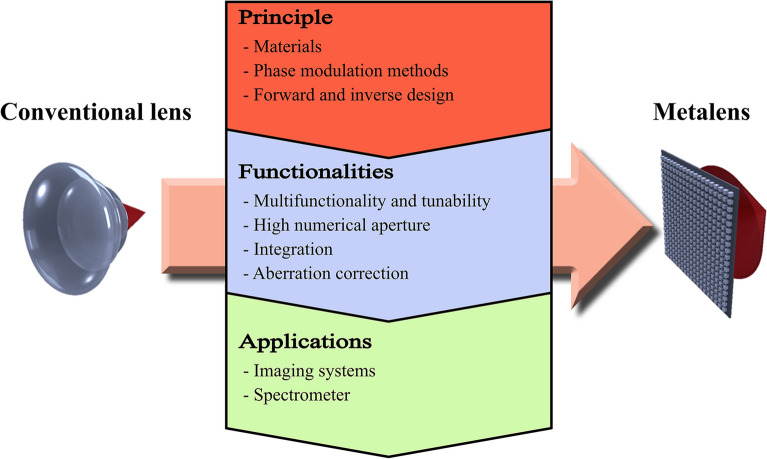

## Introduction

Metasurfaces can potentially replace existing optical components with ultrathin manners. By arranging subwavelength scatterers (i.e., meta-atoms), the characteristics of light, such as the phase [1–16], amplitude [17–20], polarization [21–28], and orbital angular momentum (OAM) [29–37], can be manipulated. Among these, studies have actively investigated a metasurface that manipulates the phase, namely, a phase gradient metasurface. Fundamental developments of the phase gradient metasurface are based on the material and phase modulation method. A meta-atom can behave like a nanoresonator, nanowaveguide, and half-wave plate, leading to a resonant phase, propagation phase, and geometric phase, respectively. The corresponding phase modulation methods change as the material transitions from a metal to a dielectric in the visible region. Implementing localized surface plasmon resonance (LSPR)-based resonant and geometric phases in a metal results in considerable ohmic loss [31, 38, 39]. By contrast, Mie-resonance-based resonant, propagation, and geometric phases can be implemented in a dielectric with low loss [40–42]. These developments show the possibility of replacing existing optical components such as beam steerers [43–51], holograms [52–62], and lenses [63–73]. A metasurface with a large number of degrees of freedom allows a beam steerer and hologram to have multiple steering angles and holographic images, respectively. On the other hand, a lens with such a large number of degrees of freedom can exhibit various functionalities such as aberration correction, extended depth of focus (DOF), high numerical aperture (NA), and varifocal characteristics. Therefore, the use of a metasurface instead of a conventional lens affords various possibilities.

Compared to conventional refractive or diffractive lenses, metasurface-based lenses (i.e., metalenses) have various advantages in terms of aberration correction, varifocal characteristics, high NA, extended DOF, and integration. Early metalenses operated at a single wavelength and had no other functions [39, 74]. Recently, however, metalenses with various functions have been developed. For example, achromatic metalenses have a drastically reduced volume owing to the replacement of the array of lenses, and high-NA metalenses afford high resolution for applications such as direct laser lithography [75], optical trapping [66, 76–79], and microscopic imaging [67]. Metalenses can be fabricated directly on existing optical systems, and they show high compatibility with fibers [76, 80–85], waveguides [86–90], and vertical cavity surface-emitting lasers (VCSELs) [91]. Overall, advancements in metalenses have been made in the order of principle, functionality, and application. For example, advances in inverse design (principle) enable a polarization-insensitive achromatic (functionality) metalens with a large aperture size to be designed; then, such a metalens can be used to realize a compact eyepiece with a reduced number of polarizers for use in virtual reality (application) [92].

In this paper, we review recent advancements in metalenses in the order of principle, functionality, and application. Section "Principles" introduces developments in the fundamental principles of metasurfaces and metalenses in terms of materials, phase modulation methods, and design methods. We describe the limitations of materials and how they were overcome in the order of metal, metal-insulator-metal (MIM), dielectric, and engineered dielectric. The materials used are highly related to the phase modulation methods. Subsequently, resonant, propagation, and geometric phases are introduced. Finally, the limitations of forward design and studies that have reduced the computation time of inverse design are described. Section "Functionalities" introduces the functionalities of metalenses, namely, multifunctionality, tunability, high NA, integration, and aberration correction. We describe multifunctional metalenses whose responses change according to the polarization, intensity, and OAM of incident light as well as high-NA and integrated metalenses. Aberrations, whether of monochromatic or chromatic type, degrade the performance of metalens, and we introduce studies that corrected these aberrations. Section "Applications" discusses the applications of metalenses to spectrometers and imaging systems, especially a near-eye display system and full-color router. Finally, Sect. "Conclusion and outlook" summarizes this review and discusses the future applications of metalenses.

## Principles

### Materials

**Fig. 1:**
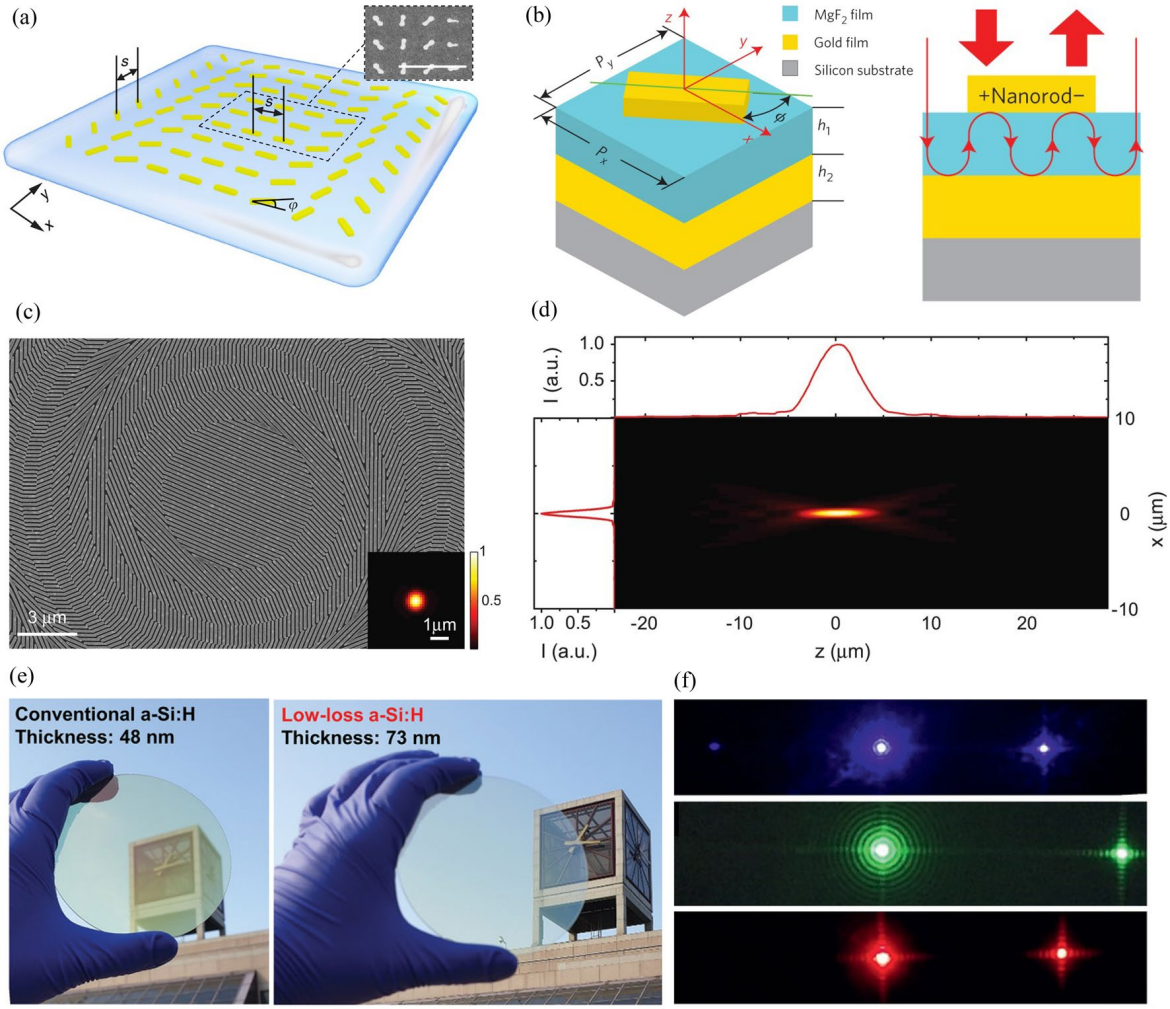
Materials for metasurfaces and metalenses. **a** Schematic of plasmonic metalens and (inset) corresponding scanning electron microscopy (SEM) image (scale bar = 1 μm). Reproduced with permission [39] (Copyright 2012, Springer Nature). **b** Structure of MIM metasurface. MgF_2_ and gold films are located below the gold nanorod, leading to Fabry-Pérot-like behavior. Reproduced with permission [93] (Copyright 2015, Springer Nature). **c** SEM image of fabricated dielectric gradient metalens. **d** Measured intensity profile of metalens in *xz*-plane. **c** and **d** are reproduced with permission [94] (Copyright 2014, American Association for the Advancement of Science). **e** Comparison of visible transparency of (left) conventional and (right) low-loss a-Si:H. **f** Captured images of beam steering by low-loss a-Si:H metasurfaces. Deflected light with wavelengths of (top) 450 nm, (middle) 532 nm, and (bottom) 635 nm are shown. (**e**) and (**f**) are reproduced with permission [95] (Copyright 2021, Wiley-VCH).

Metalenses can have various target wavelength bands depending on the application, and the materials used vary depending on the wavelength band. A complex refractive index reflects the optical properties of a material: its real part (*n*) and imaginary part (*k*) correspond to the ratio of the speed of light and absorption of light in the material, respectively. Because a higher *n* value leads to a larger modulation depth and a lower *k* value leads to a smaller absorption loss, a material with a high *n* value and a low *k* value at the target wavelength should be selected. For the visible wavelength range, the materials used for fabricating metalenses have been developed in the order of metal, MIM, dielectric, and engineered dielectric.

Chen et al. used Au to fabricate a dual-polarity metalens operating in the visible region (Fig. 1a) [39]. Because the geometric phase has an opposite sign depending on the helicity of the input light, the metalens behaves like convex and concave lens when the incident light is right circularly polarized (RCP) and left circularly polarized (LCP), respectively. A plasmonic metalens that operates in the visible region has been fabricated; however, it shows low efficiency owing to the high ohmic loss of Au and operates at a wavelength of 740 nm close to the near-infrared (NIR) region. An MIM structure with a metal layer added to the existing plasmonic metasurface has been proposed to overcome the efficiency reduction problem (Fig. 1b) [93]. This structure has a diffraction efficiency of 80%, and an efficiency of up to 50% has been measured in the 630–1050 nm broadband. However, only a reflective metasurface can be implemented owing to the presence of an additional metal plane. Therefore, the need to use a nonmetal material is increased.

A dielectric-based metasurface can have high transmittance and efficiency in the visible region. Lin et al. arranged silicon nanobeam antennas based on the geometric phase to realize a transmissive metalens operating at 550 nm, as depicted in Fig. 1c and d [94]. Different resonance features appear for each as the incident light has transverse electric (TE) and transverse magnetic (TM) polarization. An appropriate width is selected so that the phases for TE and TM differ by π. The selected nanobeam (width: 120 nm, height: 100 nm) has a high-efficiency geometric phase at a wavelength of 550 nm, and it can act as an axicon or blazed grating in addition to a lens. In addition to the advantage of lower loss in the visible region compared to that of metal, a dielectric affords the advantage of having a magnetic response of comparable strength to the electric response. To obtain a magnetic response with a metal, a complex-shaped structure like a split-ring resonator is required; by contrast, a dielectric can exhibit electric and magnetic responses even with simple structures [96]. The fact leads to the implementation of dielectric-based resonant phase metasurface (i.e., Huygens’ metasurface) with near-unity transmission [97], as discussed in the next section.

Silicon-based materials such as polycrystalline silicon (p-Si), crystalline silicon (c-Si), and hydrogenated amorphous silicon (a-Si:H) were mainly used for producing dielectric metasurfaces. However, they suffer significant absorption loss in the low wavelength range of the visible region. As alternatives, silicon nitride (Si_3_N_4_), titanium dioxide (TiO_2_), or gallium nitride (GaN) have been used in metasurfaces that operate in this region. However, Si_3_N_4_ has *n* of around 2, leading to a high height and aspect ratio. Although TiO_2_ and GaN have a higher refractive index than that of Si_3_N_4_, they are not compatible with complementary metal–oxide–semiconductors (CMOS) and require a complex process of low-temperature atomic layer deposition [42, 98] and high-aspect-ratio two-step dry etching [99, 100], respectively. To overcome these limitations, an engineered dielectric that optimizes the dielectric deposition conditions to maintain *n* as much as possible and lower *k* has been developed. Yang et al. realized a-Si:H with low loss in the entire visible range by optimizing the deposition conditions (Fig. 1e and f) [95]. They analyzed the disorder and bonding length of Si and adjusted the chemical deposition conditions to control the hydrogenation and silicon disorder, thereby lowering *k*. This low-loss a-Si:H has been used to fabricate beam steering metasurfaces for wavelengths of 450 nm, 532 nm, and 635 nm. These metasurfaces respectively exhibit measured ratios of deflected beam intensity to incident beam intensity of 42%, 65%, and 75%, all of which are much higher than that of typical silicon-based metasurfaces [56, 101, 102].

### Phase modulation

**Fig. 2:**
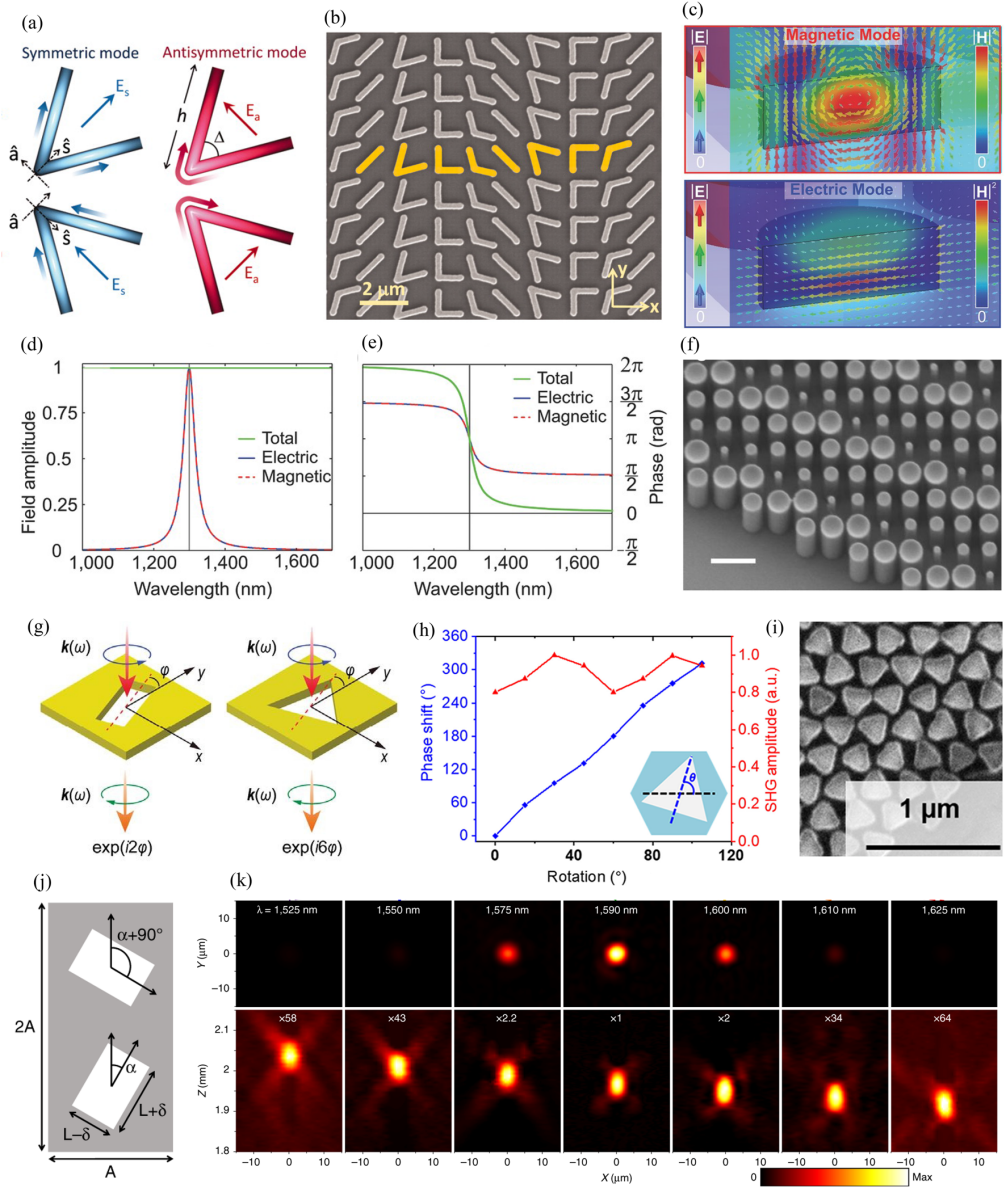
Phase modulation methods. **a** Symmetric and antisymmetric mode of V-shaped gold antenna. **b** SEM image of plasmonic resonant-phase-based metasurface. (**a**) and (**b**) are reproduced with permission [103] (Copyright 2011, American Association for the Advancement of Science). **c** (top) Magnetic and (bottom) electric mode of Mie-resonant-phase-based metasurface. **d** Field amplitude and **e** phase spectrum of spectrally overlapped electric dipole and magnetic dipole resonances. **c-e** are reproduced with permission [97] (Copyright 2015, Wiley-VCH). **f** Side-view SEM image of propagation-phase-based metalens (scale bar = 600 nm). Reproduced with permission [41] (Copyright 2016, American Chemical Society). **g** (left) Two-fold and (right) three-fold symmetric meta-atoms in a square lattice. Reproduced with permission [104] (Copyright 2021, American Physical Society). **h** Simulated phase shift and second harmonic generation amplitude versus rotation angle of nonlinear meta-atom. **i** SEM image of fabricated nonlinear metalens. (**h**) and (**i**) are reproduced with permission [105] (Copyright 2022, American Association for the Advancement of Science). **j** Schematic of p2 space group unit. **k** Experimental intensity distributions (top) on the focal plane and (bottom) along the optical axis. (**j**) and (**k**) are reproduced with permission [106] (Copyright 2022, Springer Nature)

After a material suitable for the operating wavelength range is identified, the phase is modulated by adjusting the shape, dimension, and in-plane rotation angle of the meta-atom. Phase modulation methods are typically based on the resonant phase, propagation phase, or geometric phase. Recently, an exceptional topological phase that encircles the exceptional point of a non-Hermitian metasurface has also been proposed [107]. However, it is beyond the scope of this study because it has not yet been used in a practical metalens.

The resonant phase method uses the rapid phase shift near the resonance wavelength. This method uses the general LSPR and Mie resonances. Because resonance causes a phase shift of π, the 2π phase is covered in different ways depending on the type of resonance. LSPR results from coherent oscillations of the conduction electrons on the metal surface. Yu et al. obtained a 2π phase by using a geometric phase together with a resonant phase due to LSPR [103]. A symmetric and an antisymmetric mode are induced according to the polarization of light incident on the V-shaped gold antenna (Fig. 2a), and each mode has a different amplitude and phase owing to the different resonance conditions. Because this results in light with polarization orthogonal to the incident light, the geometric phase can be used. Owing to the resonance condition, four meta-atoms with π/4 phase intervals can be obtained by adjusting the antenna length and angle between the rods. Another four meta-atoms with π phase shift are also obtained by rotating each of them by 90°. Together, these eight meta-atoms can cover the 2π phase, as depicted in Fig. 2b.

Unlike the plasmonic counterpart, the resonant phase using Mie resonance obtains a 2π phase by using both the magnetic dipole (MD) resonance and electric dipole (ED) resonance. These Mie resonance-based resonant phase metasurface has been conventionally termed as the Huygens’ metasurface, owing to the fact that the crossed electric and magnetic dipoles function analogously to a Huygens’ source [108]. Furthermore, the criteria for achieving an ideal Huygens’ source, namely the conditions of near-unity transmission and the presence of electric and magnetic dipole resonances within a single structure, can be attained by dielectric meta-atom with Mie resonances. When the wavelength is comparable to π times the object diameter, the scattering pattern by the spherical object can be obtained with the Mie solution. The multipole expansion of the scattered field can be expressed as

1 $${C}_{\text{s}}=\frac{2{\uppi }}{{k}^{2}}{\sum }_{n=1}^{{\infty }}(2n+1)({\left|{a}_{\text{n}}\right|}^{2}+{\left|{b}_{\text{n}}\right|}^{2})$$

where ${a}_{\text{n}}$ and ${b}_{\text{n}}$ are the nth-order electric and magnetic multipole, respectively, and $k$ is the wavenumber. As mentioned in the previous section, dielectrics can have electric and magnetic responses of comparable strength without complex geometries. Decker et al. implemented a resonant phase metasurface capable of 2π phase cover by using the fact that both MD and ED resonances can be induced in a silicon nanodisk (Fig. 2c) [97]. The scattering pattern in the forward direction constructively interferes when the amplitude and phase of MD and ED resonances match. At the same time, the scattering pattern in the backward direction interferes destructively, leading to near-unity transmission (Fig. 2d). Further, the π phase shifts of each resonance produce the 2π phase, as shown in Fig. 2e.

The propagation phase is modulated by locally controlling the phase retardation, with each meta-atom acting as a nanowaveguide. The phase retardation caused by the nanowaveguide can be expressed as

2 $${\phi }_{\text{p}}=\frac{2{\uppi }}{\lambda }{n}_{\text{e}\text{f}\text{f}}h$$

where ${n}_{\text{e}\text{f}\text{f}}$, $h$, and $\lambda$ are the effective index, height, and wavelength, respectively. The effective index depends on the shape and dimension of the meta-atom. To obtain a 2π phase as a propagation phase, the height versus wavelength and the difference in effective index between meta-atoms should be high. In addition, selecting an appropriate period is important because it has both upper and lower constraints. Specifically, if the period is large, an additional propagation mode appears, and the efficiency decreases owing to diffraction; further, if the period is small, the distance between meta-atoms decreases, and it can cause unwanted coupling between meta-atoms. The propagation phase is often used to impart polarization-insensitive characteristics because isotropic meta-atoms can be used; this is different from the case of the geometric phase (Fig. 2f) [41].

When circularly polarized (CP) light passes through an in-plane rotated anisotropic meta-atom, the light converted to orthogonal polarization gets an additional phase equal to two times the rotation angle. The resulting electric field can be expressed as

3 $${E}_{\text{t}}=\text{R}\left(-\theta \right)\left[\begin{array}{cc}{t}_{\text{l}}& 0\\ 0& {t}_{\text{s}}\end{array}\right]\text{R}\left(\theta \right)\left[\begin{array}{c}1\\ \pm i\end{array}\right]=\frac{{t}_{\text{l}}+{t}_{\text{s}}}{2}\left[\begin{array}{c}1\\ \pm i\end{array}\right]+\frac{{t}_{\text{l}}-{t}_{\text{s}}}{2}{e}^{\mp i2\theta }\left[\begin{array}{c}1\\ \mp i\end{array}\right]$$

where ${E}_{\text{t}}$, $\text{R}\left(\theta \right)$, and $\theta$ are the transmitted electric field, rotation matrix, and in-plane rotation angle, respectively, and ${t}_{\text{l}}$ and ${t}_{\text{s}}$ are the transmission coefficients along the long and short axes, respectively. In general, this geometric phase corresponds to $2\theta$; however, it corresponds to $\text{n}\theta$ in the cases of metasurfaces with high-order rotational symmetric meta-atoms, nonlinear response, and quasi-bound states in the continuum (quasi-BIC)-based nonlocal response. Notably, even structures with more than three-fold rotational symmetry can cover the 2π phase owing to the rotation of the effective principal axis (Fig. 2g) [104]. For n-fold rotational symmetric structures on the square lattice, the geometric phase is $\pm 2\text{n}\theta$ and $\pm \text{n}\theta$ when n is odd and even, respectively. The nonlinear metasurfaces also have an anomalous geometric phase of $(\text{m}+1)\theta$, where m is the nonlinear order. Figure 2h and i show the demonstrated second harmonic generation nonlinear metalens with a three-fold symmetric meta-atom [105]. Quasi-BIC-based nonlocal metasurfaces can modulate the phase only near the resonance wavelength, and they have a phase corresponding to $4\theta$ [106, 109]. The p2 space group composed of two anisotropic structures with rotation angles of $\theta$ and $\theta$+90° is the unit cell of the nonlocal metasurface, as depicted in Fig. 2j. In the incident CP light, only the linearly polarized (LP) light with a polarization angle of $2\theta$ couples to the unit cell and excites the corresponding mode. This process involves the additional phase of $2\theta$, and the conventional geometric phase of $2\theta$ is added in the out-couple process, resulting in the phase of $4\theta$. In addition, this phase can be $\text{n}\theta$ when different types of lattices are used. Because this phase is applied only when the corresponding mode is excited, phase modulation occurs only near the resonance wavelength. Figure 2k shows a nonlocal metalens with a lens function at the resonance wavelength of 1590 nm; this lens function disappears abruptly at other wavelengths.

### Forward and inverse design

**Fig. 3:**
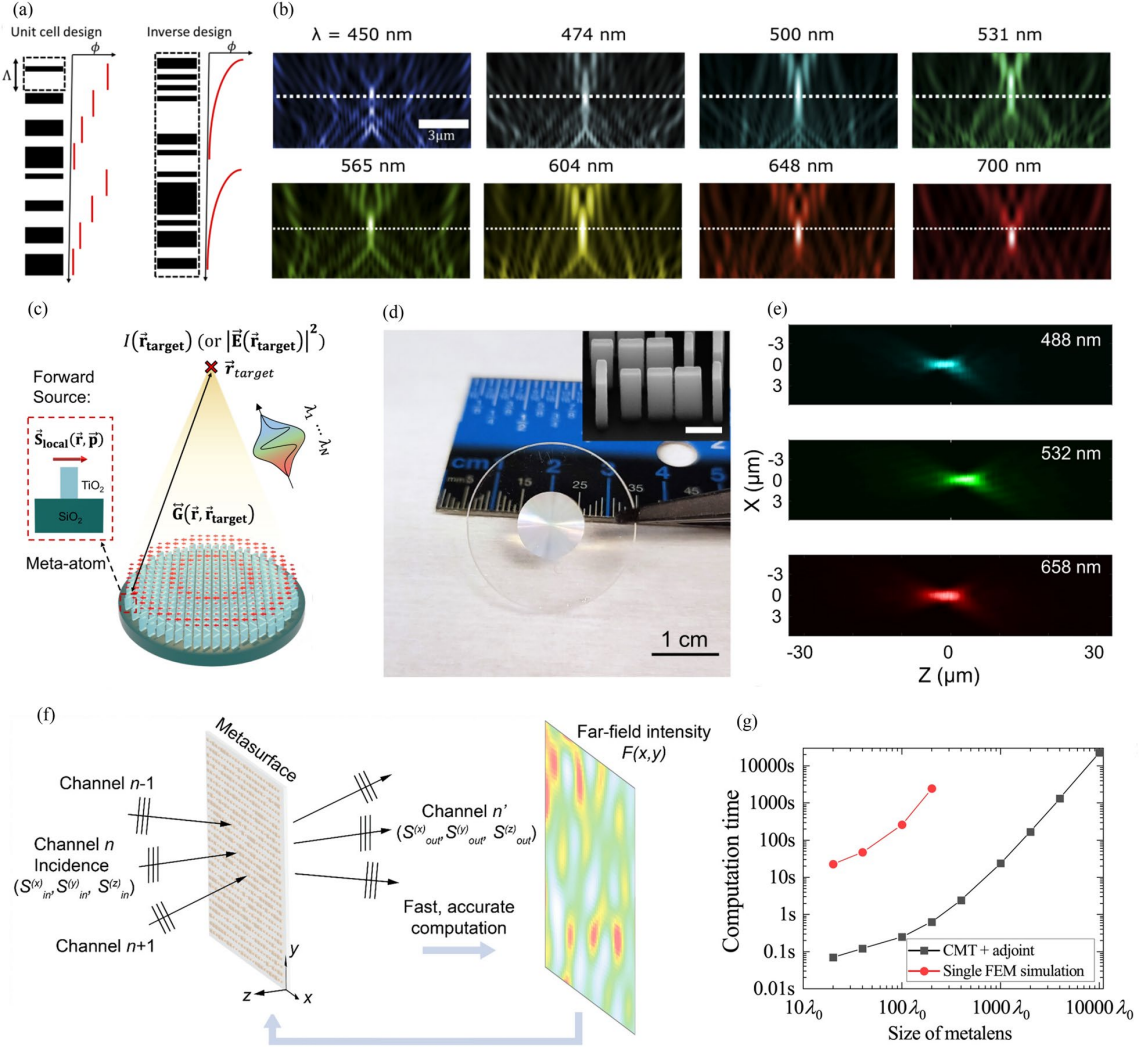
Inverse design of metalenses. **a** Comparison between (left) unit cell-based design and (right) inverse design. **b** Normalized intensity profile on longitudinal plane of inverse-designed high-NA achromatic metalens. Dotted lines indicate focal plane of metalens. (**a**) and (**b**) are reproduced with permission [110] (Copyright 2020, Optical Society of America). **c** Surrogate-model-based fast approximate solving of forward simulation. **d** Photograph of fabricated metalens with a diameter of 1 cm and achromatic and polarization-insensitive functionalities. (inset) SEM image of metalens (scale bar = 500 nm). **e** Experimental intensity distribution on *xz*-plane for wavelengths of (top) 488 nm, (middle) 532 nm, and (bottom) 658 nm. **c**-**e** are reproduced with permission [92] (Copyright 2022, Springer Nature). (f) Schematic of coupled-mode theory (CMT)-based forward simulation model. **g** Comparison of computation time between (red) single finite-element method simulations and (black) one iteration using CMT approach. **f** and **g** are reproduced with permission [111] (Copyright 2021, American Chemical Society)

After identifying the phase modulation method suitable for the given application, a metalens can be designed using the phase map derived through the lens equation. This phase map can be expressed as

4 $$\phi \left(x,y\right)= -\frac{2{\uppi }}{\lambda }(\sqrt{{x}^{2}+{y}^{2}+{f}^{2}}-f)$$

where $\lambda$ and $f$ are the wavelength and focal length, respectively, and $x$ and $y$ are the *x*- and *y*-directional position on the metalens, respectively. Metasurfaces can be used to create arbitrary wavefronts by adjusting the phase locally. Therefore, they do not suffer from spherical aberrations when using a phase map without spherical aberrations. However, chromatic aberration still exists because Eq. (4) depends on the wavelength.

Metalenses can be forward-designed by repeating the process of forward simulation and scattered field confirmation to achieve the desired performance. Specifically, forward design involves the following process: selection of material and phase modulation method, repetition of forward simulation, derivation of phase map, and arrangement of meta-atoms. First, a material and a phase modulation method that are suitable for the operating wavelength and application purpose, respectively, are selected. Then, the forward simulation is repeated under periodic boundary conditions, and the parameters of the meta-atom are optimized to obtain the desired optical characteristics. Local electromagnetic responses can be obtained under periodic boundary conditions owing to the locally periodic approximation. The locally periodic approximation is applicable for moderate NA, in which adjacent meta-atoms do not change rapidly [92, 112, 113]. The meta-atom obtained under this condition acts locally and can be treated as an independent pixel. An appropriate focal length can be known through the target NA and diameter of the metalens, and the corresponding phase map is obtained by substituting it into Eq. (4). Finally, a metalens can be designed by arranging the previously obtained meta-atoms according to the obtained phase map.

Large area, aberration correction, and high NA are the desired functionalities for realizing the widespread use of metalenses. Achieving these functionalities requires a large number of degrees of freedom; however, they can be optimized to only a limited extent through forward design. In addition, the existing unit-cell-based approach is limited by a theoretical upper bound of efficiency [110]. Inverse design has emerged recently as a means to overcome these limitations. Metalenses can be inverse-designed by defining the desired function as a figure of merit (FoM) and optimizing the black box region to maximize the FoM. The inverse design method evaluates the FoM by altering each degrees of freedom and iteratively advancing in the direction of its increase. The inverse design method can be categorized into two groups: periodic and non-periodic, based on the defined degrees of freedom. The periodic method involves inverse designing while maintaining periodicity, similar to conventional metasurfaces. This is accomplished by designing a phase map [114] or a single meta-atom shape [115]. For instance, in the case of inverse design of phase map, the meta-atom’s dimension is predetermined based on the conversion efficiency, followed by designing the entire metasurface by arranging the meta-atoms with the designed phase map. On the other hand, the non-periodic method doesn’t exhibit periodicity and includes methods such as topology optimization post blurring of a pre-designed metasurface [116] or adjusting the permittivity of tiny pixels [110]. The blurring method offers a good initial value for inverse design since setting the proper initial values is crucial. While the unit-cell-based approach has theoretical efficiency limits, non-periodic methods can overcome these limits. These methods set the black box region for the entire area of the metalens; however, a large-area forward simulation in the inverse design process incurs high computational costs. Therefore, many efforts have been made recently to lower these computational costs. For example, studies have used the adjoint method [110], surrogate model [92], and CMT [111]. The adjoint method uses the reciprocity of Green’s function to obtain the gradient of the FoM with only two simulations per iteration. The inverse design of a high-NA achromatic metalens has been demonstrated using this approach [110]. Freeform and constant-*z* (i.e., constant permittivity in the direction of the optical axis) metalenses have been designed, and a constant-*z* high-NA (NA = 0.9) metalens shows broadband (450–700 nm) achromatic operation, as depicted in Fig. 3(a) and 3(b). The computational cost incurred for inverse design can be reduced by speeding up the forward simulation with a surrogate model (Fig. 3(c)) [92]. A Chebyshev-interpolation-based surrogate model can rapidly predict the local field for an arbitrary meta-atom. A metalens with a large area (diameter: 1 cm), achromatic, and polarization-insensitive functionalities has been implemented by this approach, as shown in Fig. 3(d) and 3(e). CMT can also speed up the forward simulation [111]. The output electromagnetic response is obtained by calculating the coupling between incident free-space modes and the fundamental TE modes of each meta-atom (Fig. 3(f)). The proposed CMT model is several times faster than finite-element method simulations. As a result, a high-NA (NA = 0.9) metalens can be designed rapidly by inverse design using the CMT model and adjoint optimization (Fig. 3(g)).

## Functionalities

### Multifunctionality and tunability

**Fig. 4:**
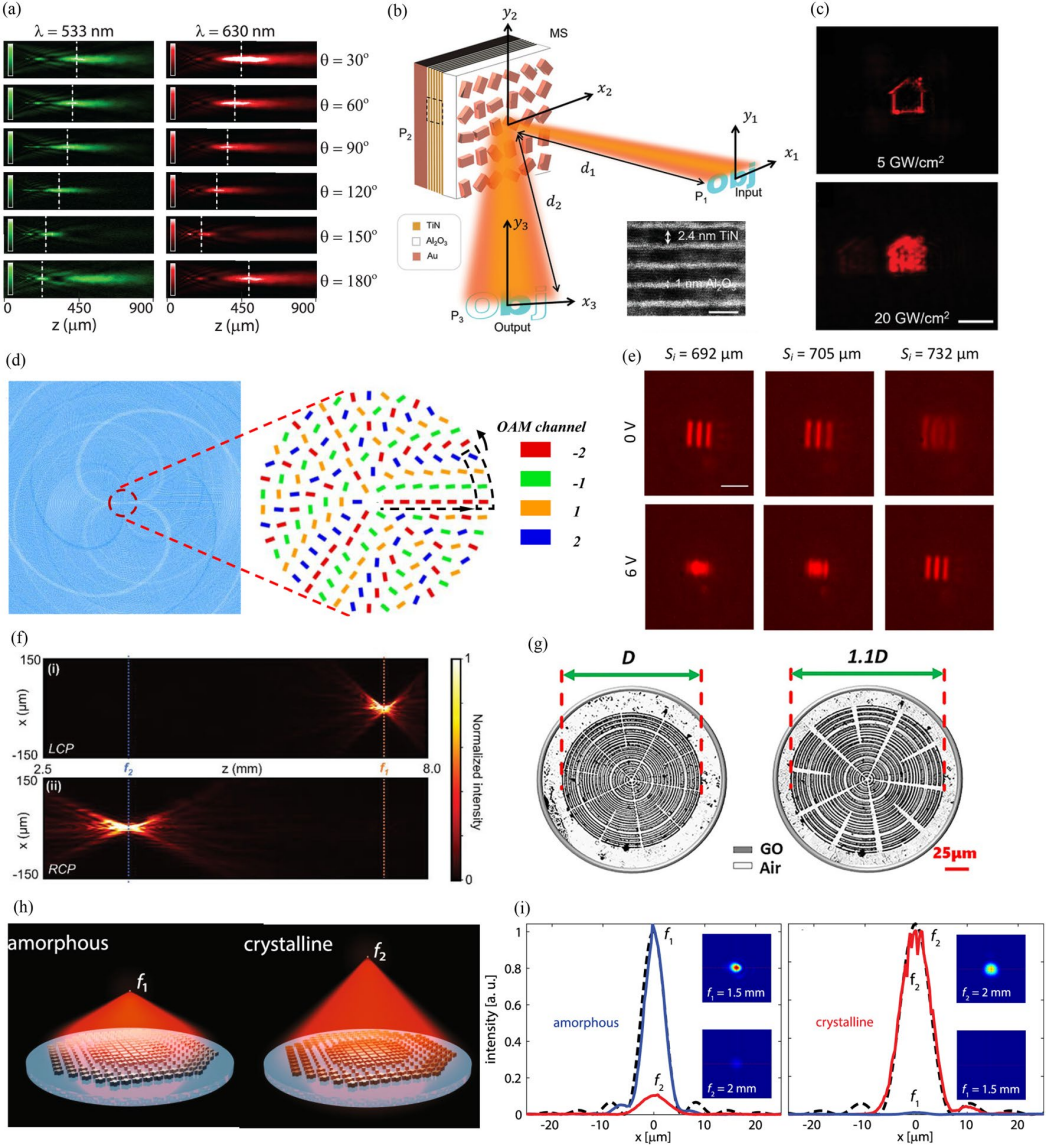
Multifunctional and tunable metalenses. Multifunctional metalenses: **a** Measured normalized intensity distribution on *xz*-plane of varifocal metalens. *θ* represents polarization angle of incident light. Reproduced with permission [63] (Copyright 2019, American Chemical Society). **b** Schematic of nonlinear intensity multifunctional metalens. (inset) Transmission electron microscopy image of metallic quantum wells (scale bar = 5 nm). **c** Captured images for incident light intensity of (top) 5 GW/cm^2^ and (bottom) 20 GW/cm^2^ (scale bar = 200 μm). (**b**) and (**c**) are reproduced with permission [117] (Copyright 2022, Wiley-VCH). **d** Interleaving method for multiplexing multiple OAM modes in metalens. Reproduced with permission [118] (Copyright 2022, Springer Nature). Tunable metalenses: **e** Imaging experiment results of varifocal metalens for varying applied voltages. Voltages of 0 and 6 V produced clear images at distances of 692 and 732 μm, respectively (scale bar = 10 μm). Reproduced with permission [64] (Copyright 2018, American Chemical Society). **f** Experimental intensity profile of bifocal metalens on *xz*-plane for (top) LCP and (bottom) RCP incident light. Reproduced with permission [119] (Copyright 2021, Wiley-VCH). **g** Stretching condition of graphene-oxide-based metalens. This metalens is stretched uniformly, and the stretch ratio is 1.1 times. Reproduced with permission [120] (Copyright 2021, American Chemical Society). **h** Focal length change of GSST-based metalens. This metalens has different focal lengths in amorphous and crystalline states. **i** Measured focal spot profiles for (left) amorphous and (right) crystalline states. (inset) Captured images of focal spots. **h** and **i** are reproduced with permission [121] (Copyright 2021, Springer Nature)

Metalenses are advantageous compared to conventional refractive or diffractive lenses because multiple functionalities can be easily incorporated in them. Multifunctional metalenses whose response depends on the polarization, intensity, and OAM of incident light have recently been demonstrated. Polarization-multifunctional metalenses have been actively studied for both CP and LP light. Tian et al. combined the geometric and propagation phases to realize a bifocal metalens in which the focal length varies depending on LCP and RCP light [122]. When the meta-atom rotates, LCP and RCP light have geometric phases with opposite signs; however, the propagation phases are the same. Incident LCP and RCP light are focused at a distance of 10 and 13 μm, respectively, and the relative intensity of the two focal spots can be adjusted by changing the ellipticity of polarization. Aiello et al. used an asymmetric meta-atom to realize a metalens with different focal lengths for *x*- and *y*-polarized incident light [63]. Because the propagation phase differences of *x*- and *y*-polarized light appear differently when the width and length of the nanopost are changed, different phase maps for the two polarizations are possible. The focal length of the demonstrated varifocal metalens continuously changes as the polarization angle of the LP light changes (Fig. 4(a)).

The response of the metalens can be adjusted by varying the intensity and OAM of incident light. A nonlinear metallic quantum well layer is inserted into the insulator layer of the MIM structure to realize an intensity-multifunctional metalens (Fig. 4b) [117]. The phase retardation between the long and short axes of the nanopost can be controlled by the incident intensity owing to the Kerr effect. The implemented metalens uses two lens phases with a lateral offset, and it shows an edge detection image and a full image at intensities of 5 GW/cm^2^ and 20 GW/cm^2^, as shown in Fig. 4(c). Light with an azimuthal phase dependence of ${e}^{il\varphi }$, where $l$ and $\varphi$ are the topological charge and azimuthal angle, respectively, has an OAM of per photon [123–125]. $l$can have an infinite number of values, and each state is orthogonal to the other. Consequently, many functions can be added beyond the bifunctional when using the OAM as a degree of freedom. A multiband varifocal metalens whose focal length changes according to the OAM modes of $l$ = ±1, ± 2 at wavelengths of 532 and 633 nm has recently been demonstrated [118]. A spiral phase corresponding to each OAM mode is obtained and combined in an interleaved manner to achieve the varifocal function (Fig. 4(d)). The implemented metalens has focal lengths of 5.268–35.126 mm (532 nm) and 4.532–29.048 mm (633 nm) as $l$ changes from − 2 to 2. The characteristics of the incident light can be varied in a laboratory; however, doing so is not easy. Therefore, the need for tunable metalenses with varying responses depending on external stimuli, such as electrical or mechanical stimuli, has emerged.

Tunable metalenses can be classified into those that tune the external environment and those that tune the meta-atom itself. Afridi et al. placed polydimethylsiloxane (PDMS) and a spiral gold heater at the bottom of a substrate to control the phase retardation by adjusting the refractive index of PDMS according to the temperature change [64]. The metalens on the top of the substrate has a varifocal characteristic in that its focal length corresponds to the applied voltage of the heater (Fig. 4e). A nanopillar made of amorphous silicon works using the principle of Huygens’ metasurface, and metalenses with focal lengths of 600 and 1000 μm are designed, respectively. In each metalens, the focal length changes by 15% (600 μm) and 23% (1000 μm) at 12 V. The polarization of the incident light can also be changed by adjusting the external environment. Badloe et al. designed a metalens with different focal lengths for LCP and RCP light using both geometric and propagation phases [119]. Combining the metalens with an electrically controllable liquid crystal (LC) that can alter the polarization of the incident light produces the electrically tunable bifocal functionality. LC produces LCP light at 1.1 V with a corresponding focal length of 7.5 mm and RCP light at 1.3 V with a corresponding focal length of 3.7 mm (Fig. 4(f)).

The active control of the metalens itself can be achieved through a mechanical stimulus by using a stretchable substrate or changing the refractive index by varying the phase of the material. Wei et al. implemented a stretchable varifocal metalens by using mechanically robust graphene oxide (GO) [120]. Stretching a metalens increases the distance between the GO rings, thereby causing its focal length to change (Fig. 4g). They showed that the metalens could have a focal length tuning range of more than 20% for red, green, and blue light. Shalaginov et al. demonstrated a varifocal metalens by using the fact that the refractive index varies as the phase of Ge_2_Sb_2_Se_4_Te_1_ (GSST) changes from amorphous to crystalline (Fig. 4h) [121]. GSST has broadband transparency in the infrared regime for both amorphous and crystalline states, unlike conventional Ge_2_Sb_2_Te_5_ (GST). They designed 16 types of meta-atoms that satisfy desired phase retardations in both the amorphous and crystalline states. The nonmechanical method had limitations in terms of the optical quality. However, in the present study, a record-high switching contrast ratio of 29.5 dB was realized with diffraction-limited performance (Fig. 4i). Although many limitations need to be overcome in tunable metalenses, such as switching speed and efficiency, various tuning mechanisms and their developments have raised expectations for the implementation of an ideal tunable metalens.

### High NA

**Fig. 5:**
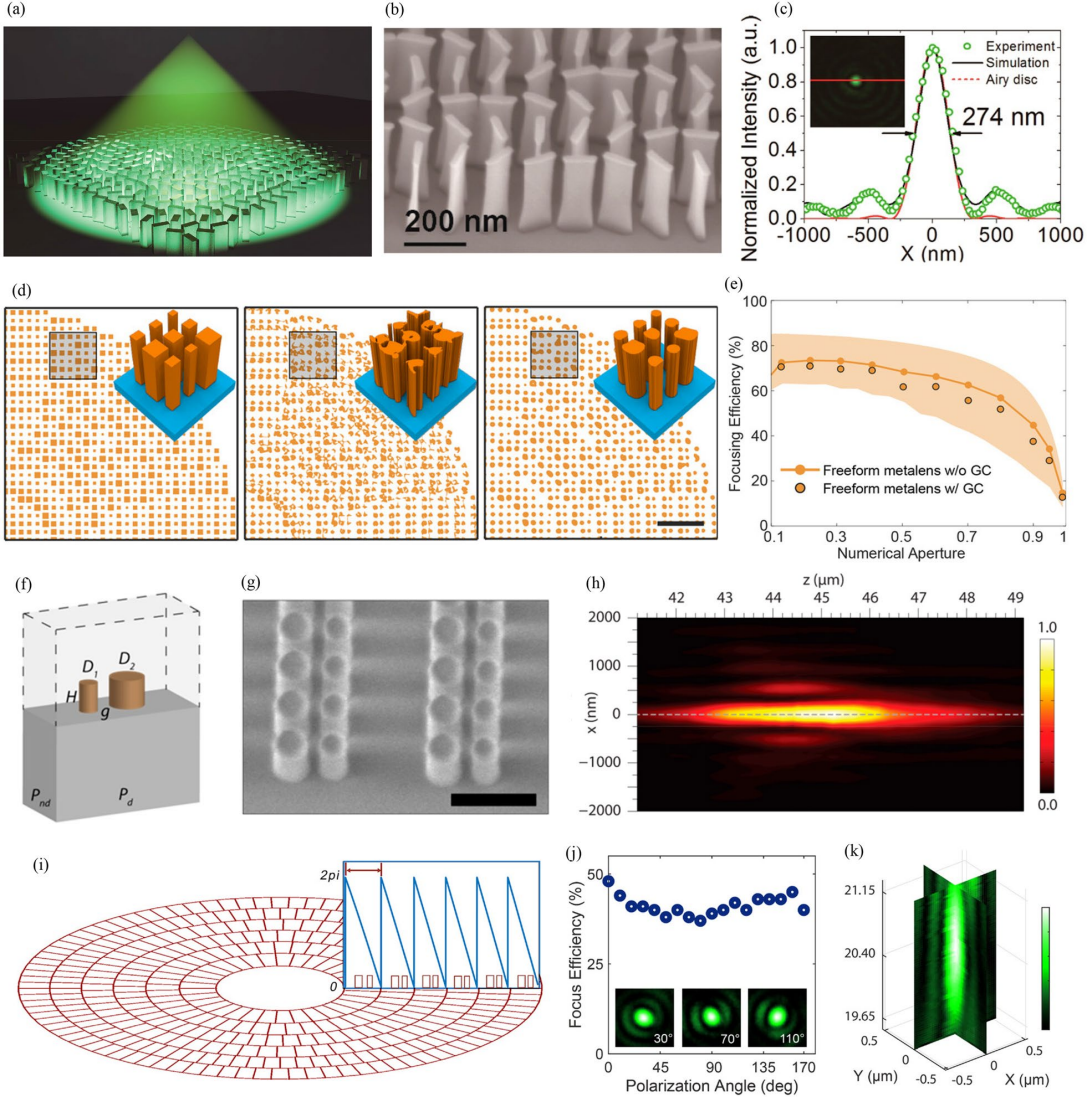
High-NA metalenses. Optimization-based ultrahigh-NA metalenses: **a** Schematic of metalens designed using hybrid optimization algorithm. **b** Side-view SEM image of metalens. **c** Measured, simulated, and ideal intensity profiles of focal spot. Captured image of focal spot (inset). **a**-**c** are reproduced with permission [126] (Copyright 2018, American Chemical Society). **d** Structures of (left) conventional, (middle) topology-optimized, and (right) topology-optimized fabricable metalens (scale bar = 2 μm). **e** Simulated focusing efficiency of metalenses. Shaded area shows the region between the efficiency of conventional metalens and vector diffraction theory. (**d**) and (**e**) are reproduced with permission [116] (Copyright 2022, Wiley-VCH). Asymmetric-dimer-grating-based ultrahigh-NA metalenses: **f** Asymmetric dimer array and **g** side-view SEM image of metalens (scale bar = 500 nm). **h** Measured normalized intensity distribution on *xz*-plane. **f**-**h** are reproduced with permission [127] (Copyright 2018, American Chemical Society). **i** Adaptively arranged asymmetric dimers. Blue box shows Fresnel zones of metalens. **j** Focusing efficiency with different polarization angles. (insets) Point spread function for polarization angles of (left) 30°, (middle) 70°, and (right) 110°. **k** Three-dimensional image describing focusing performance of metalens. **i**-**k** are reproduced with permission [67] (Copyright 2022, Wiley-VCH)

A high-NA lens can be used to realize an optical system with high resolution. Metalenses with NAs of 0.6, 0.85, and 0.88 have been applied to a transmissive eyepiece [128], direct laser lithography [75], and optical trapping [76], respectively. Recently, ultrahigh-NA (NA > 0.9) metalenses have been demonstrated; these have been designed using optimization-based or asymmetric-dimer-grating-based methods. By using a hybrid optimization algorithm, Liang et al. produced an ultrahigh-NA metalens with an NA of 0.98 and focusing efficiency of 67% (Fig. 5a and b) [126]. This algorithm contains four different optimization algorithms: differential evolution, genetic algorithm, particle swarm optimization (PSO), and adaptive simulated annealing. It is used to optimize the parameters of the meta-atom, such as the length, width, height, and period. Experiments indicate that the focal spot of the metalens has a full-width at half-maximum (FWHM) of 274 nm; this agrees with the simulation result (277 nm) and Airy disk (279 nm) (Fig. 5c). Wang et al. designed a polarization-insensitive ultrahigh-NA metalens by using a cylindrical structure instead of a cuboid [66]. An NA of 0.97 was obtained using the PSO method. Optimization-based inverse design enables the design of a freeform metasurface. For example, Sang et al. recently implemented an ultrahigh-NA freeform metalens (Fig. 5d) [116]. They calculated the theoretical limitation of metalens efficiency based on vector diffraction theory, and they found that topology optimization enables the maximization of the efficiency. The designed metalens has an NA of 0.95 and focusing efficiency of 34.2% that is close to the theoretical limit of 44% (Fig. 5e). Even when the lens is designed with geometric constraints according to fabrication requirements, it has a high focusing efficiency of 29.1%. However, optimization-based design methods have disadvantages such as high difficulty of fabrication owing to small period, high aspect ratio, or complex shape.

The asymmetric-dimer-grating-based method can realize an ultrahigh-NA metalens with low fabrication difficulty. The center part of the metalens does not suffer from efficiency problems because the required steering angle of light is small. However, the outer part should steer light with a large angle; in particular, the maximum angle is 71.8° for a nonimmersed ultrahigh-NA (NA = 0.95) metalens. To solve this problem, asymmetric dimer gratings are used on the outer part of the metalens. Paniagua-Dominguez et al. covered bending angles of 0°–82° by adjusting the period of the asymmetric grating and the number of nanoantennas per period (Fig. 5f) [127]. For an angle of 55°–82°, only the period is controlled, and for an angle of 54° or less, the number of nanoantennas is also controlled. The fabricated metalens had an ultrahigh-NA of 0.99 (Fig. 5g and h). Zhang et al. implemented a metalens with an NA of 1.48 (oil-immersed) using a propagation-phase-based meta-atom with a period of 180 nm for the center part and an asymmetric dimer grating for the outer part [67]. They arranged the dimers adaptively so that each dimer functions as a single Fresnel zone (Fig. 5i). The fabricated metalens has a focusing efficiency of 42% (Fig. 5j and k), and it can be applied to confocal scanning microscopic imaging.

### Integration

**Fig. 6:**
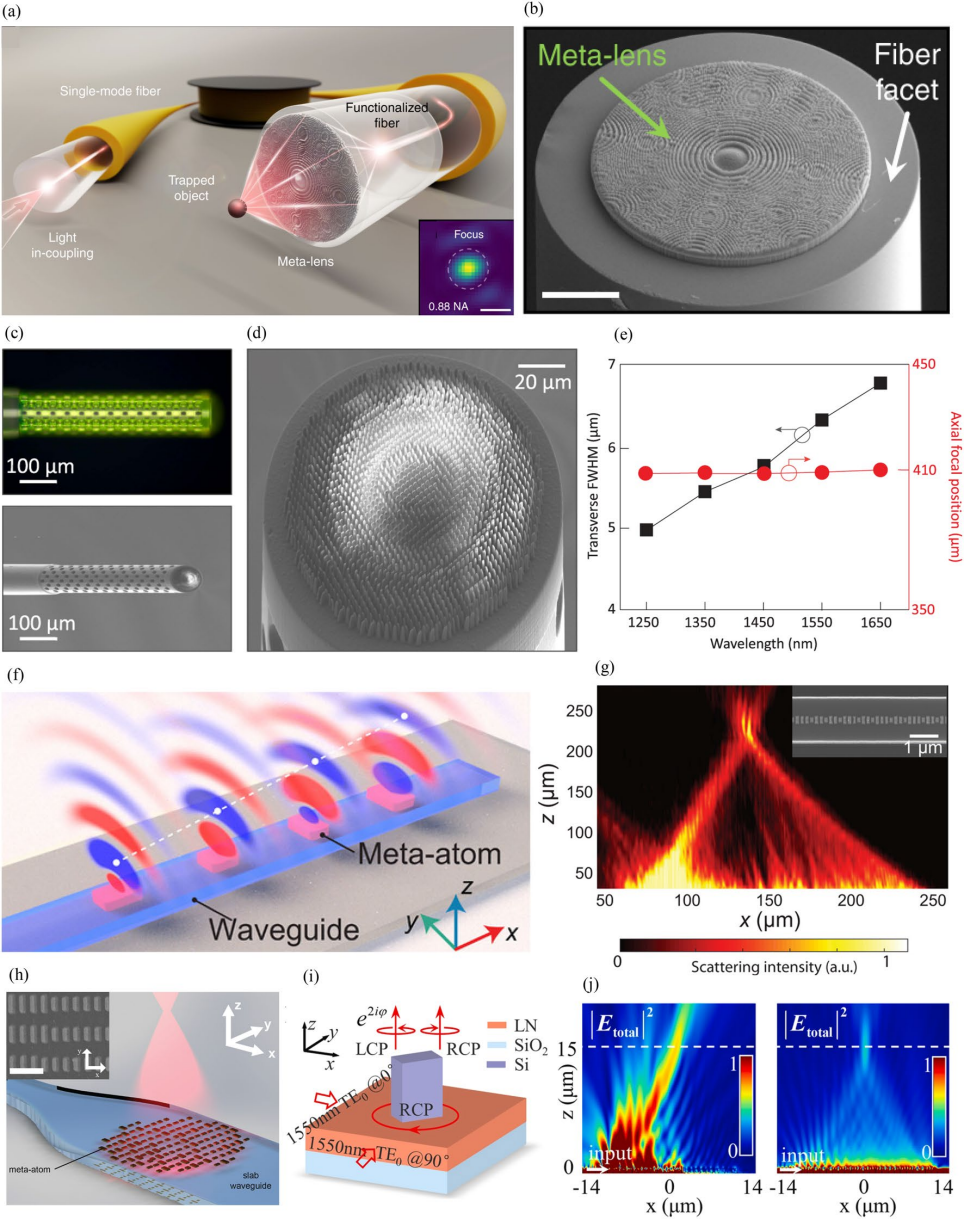
Fiber- and waveguide-integrated metalenses. Fiber-integrated metalenses: **a** Schematic of a high-NA metafiber system. (inset) Measured focal plane in water (scale bar = 500 nm). **b** SEM image of integrated metalens (scale bar = 25 μm). (**a**) and (**b**) are reproduced with permission [76] (Copyright 2021, Springer Nature). **c** (top) Optical and (bottom) SEM images of achromatic metafiber. (d) SEM image of fabricated metalens on fiber end face. (e) Measured transverse FWHM and axial focal positions at different wavelengths. **c**-**e** are reproduced with permission [80] (Copyright 2022, Springer Nature). Waveguide-integrated metalenses: **f** Waveguide-integrated one-dimensional resonance-phase-based metalens. **g** Experimental intensity distribution of metalens. Field-emission SEM image of waveguide and metalens (inset). **f** and **g** are reproduced with permission [86] (Copyright 2020, American Association for the Advancement of Science). **h** Waveguide-integrated two-dimensional metalens. (inset) SEM image of metalens (scale bar = 500 nm). Reproduced with permission [88] (Copyright 2022, American Chemical Society). **i** Waveguide-integrated metalens based on geometric phase. **j** Simulated intensity distribution above one-dimensional metalens with (left) resonant phase and (right) geometric phase. **i** and **j** are reproduced with permission [87] (Copyright 2021, De Gruyter)

A metalens can be fabricated directly onto other optical elements. This characteristic enables the integration of metalenses with fibers [76, 80], waveguides [86–88], VCSELs [91], etc. In particular, flexible optical trapping, fiber-optic communication, or endoscopic imaging can be realized when a metalens is fabricated on the tip of a fiber. Plidschun et al. demonstrated optical trapping by fabricating an ultrahigh-NA metalens on the tip of a functionalized fiber (Fig. 6a) [76]. After increasing the adhesion through the silanization process on the fiber tip, they fabricated a metalens by using a direct laser writing system, as shown in Fig. 6b. The 660-nm-wavelength light passing through the single-mode fiber is expanded as much as the metalens aperture size through the spliced multimode fiber, and it is focused through the metalens at the end of the fiber. Ren et al. fabricated an achromatic metalens on a single-mode fiber end face to solve the strong chromatic aberration caused by a conventional end face lens [80]. They printed a hollow tower on the end of the single-mode fiber for beam expansion and fabricated the metalens using two-photon polymerization (Fig. 6c and 6(d)). A thin spacer layer (thickness: 15 μm) is placed between the hollow tower and the metalens; however, it causes negligible transmittance loss. The implemented metafiber showed achromatic and polarization-insensitive focusing over the telecommunication band of 1.25–1.65 μm, as depicted in Fig. 6e.

Conventional waveguide systems, such as grating-coupler-based ones, have limitations in controlling out-coupled light. This limitation can be overcome by integrating a metalens on top of the waveguide. These waveguide-integrated metalenses can have a resonant phase or geometric phase. Guo et al. fabricated an Au-SiO_2_-Au nanoantenna-based one-dimensional metalens on a silicon waveguide to achieve off-chip light focusing (Fig. 6f and g) [86]. The guided wave is out-coupled, and directional radiation is generated when the evanescent tail of the TE_00_-guided mode excites the electric and magnetic dipolar resonance of the nanoantenna. Further, owing to the resonant phase, the 2π phase is covered by adjusting the length and width of the nanoantenna. A two-dimensional metalens can also be implemented using the same method, as shown in Fig. 6h [88]. However, this resonant phase method is sensitive to fabrication errors and makes it difficult to obtain a uniform amplitude. Fang et al. proposed the geometric-phase-based method to solve this problem [87]. They obtained CP light using propagated TE_0_ guided waves in the x and y directions; the phase difference between them was calculated as π/2 and -π/2 for LCP and RCP light, respectively (Fig. 6i). One-dimensional metalenses with resonant- and geometric-phase-based methods are designed and compared as a proof-of-concept. The resonant- and geometric-phase-based metalens asymmetrically and symmetrically focused light owing to the amplitude deviation, respectively (Fig. 6j). This result highlights the amplitude uniformity of the geometric-phase-based method; however, the low efficiency (simulated efficiency of 10%) remains a problem.

### Aberration correction

A bundle of rays from an object point passes through the lens and forms an image point on the image plane. In an ideal lens, the spot size converges to 0 on the image plane; however, most lenses have a finite spot size because of the existence of aberrations. When spherical aberration, coma aberration, and astigmatism increase, a large spot is created, and the image quality deteriorates. Further, if the distortion is large, the magnification for each field differs, and the image takes a shape that is different from that of the real object. All of these are major problems in an imaging system. Therefore, such aberrations must be compensated. Aberrations are classified as monochromatic or chromatic aberrations, as discussed below. In a conventional lens, aberrations can be compensated effectively by using a compound lens or aspherical lens. However, the resulting bulky size of the lens limits its applications. By contrast, a metalens is relatively very compact, and therefore, many studies are exploring its use for aberration compensation.

#### Monochromatic aberration

**Fig. 7:**
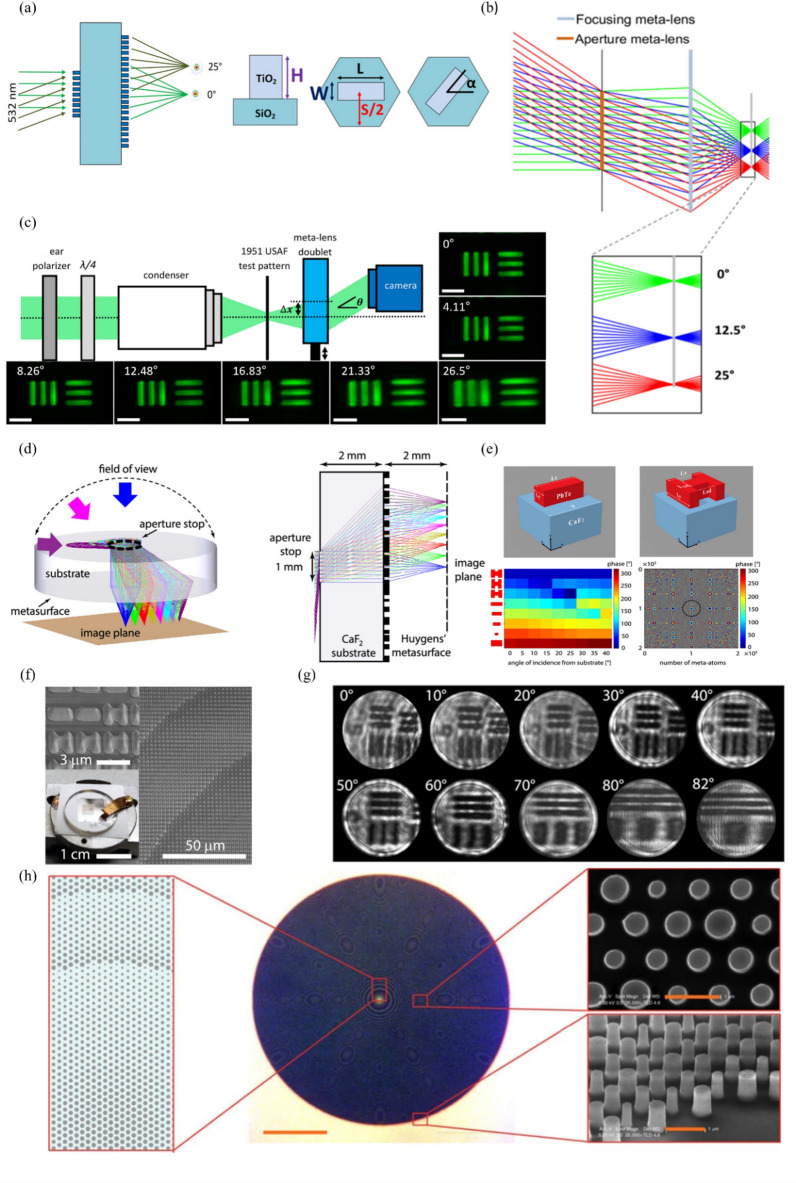
Monochromatic aberration compensation lens: **a** Schematic of metalens doublet. A metalens is present on both sides of the glass substrate. The meta-atoms of each lens have the same width, height, and length but have different angles. **b** Light incident from different angles forms images on the same image plane, with aberrations being compensated by an aperture metalens and a focusing metalens. **c** Schematic diagram of metalens doublet’s imaging setup and images for each angle of incidence (scale bar: 11 μm). **a**-**c** are reproduced with permission [129] (Copyright 2017, American Chemical Society). **d** Schematic diagram of an ultrawide-angle lens with an aperture and a metalens. **e** (top) Tilted view of a rectangular and an H-shaped meta-atom. (bottom left) Phase delay according to angle of incidence for each meta-atom shape. (bottom right) Metalens phase profile, where the black dashed circle indicates the aperture stop position and size. **f** SEM images of fabricated metalens. **g** Projected images of the 1951 USAF resolution test target with a period of 13.9 μm. **d**-**g** are reproduced with permission [130] (Copyright 2020, American Chemical Society). **h** Schematic and SEM images of polarization-insensitive metalens (scale bars: (center) 100 μm and (top- and bottom-right) 1 μm. Reproduced with permission [131] (Copyright 2015, Springer Nature)

The monochromatic aberration changes according to the field of view and NA. In general, the monochromatic aberration converges to zero in a paraxial optical system; however, aberration occurs because a real lens has a finite size. Therefore, proper aberration compensation is required to obtain high-quality images. Monochromatic aberrations are usually described by the five Seidel aberrations, as described below (Eqs. 5–9).

5 $${C}_{\text{0,4},0}{r}^{4}:spherical\,aberration$$

6 $${C}_{\text{1,3},1}\sigma {r}^{3}\text{c}\text{o}\text{s}\varphi:coma\,aberration$$

7 $${C}_{\text{2,2},2}{\sigma }^{2}{r}^{2}{\text{c}\text{o}\text{s}\varphi }^{2}:astigmatism\,aberration$$

8 $${C}_{\text{2,2},0}{\sigma }^{2}{r}^{2}:field\,curvature$$

9 $${C}_{\text{3,1},1}\sigma r\text{c}\text{o}\text{s}\varphi:distortion$$

$$\sigma = \frac{\eta }{{\eta }_{\text{m}\text{a}\text{x}}}(0\le \sigma \le 1)$$,

10 $$r= \frac{\rho }{{\rho }_{\text{m}\text{a}\text{x}}}(0\le \rho \le 1)$$

  

where ${C}_{\sigma , r, \text{c}\text{o}\text{s}\varphi }$, $\eta$, $\rho$, and $\text{c}\text{o}\text{s}\varphi$ are the coefficient of aberration, image height, radial coordinate on reference sphere, and angle of rotation from *y*-axis on a reference sphere, respectively. A spherical aberration is caused by $r$ and is proportional to NA and lens diameter. Such aberrations need to be compensated because they are strongly related to the quality of the central part of the image. A coma aberration is affected by $\sigma$, $r$, and $\text{c}\text{o}\text{s}\varphi$, and it generally increases as the field of view becomes wider. At the off-axis of the image, the shape of a spot becomes similar to that of a comet, resulting in image quality deterioration. Astigmatism is an aberration that causes the focus of sagittal and tangential rays from off-axis object points to form elsewhere. Like coma aberration, it occurs in the corner of the image rather than at the center. A field curvature represents the difference between the focus position in the center of the image and the focus position in the periphery. Therefore, if the field curvature is large, the periphery is blurred when focusing on the center, and the center is blurred when focusing on the periphery. Finally, distortion does not affect the image quality; however, the image does appear distorted. A small distortion can be compensated through software correction; however, a large distortion can only be corrected to a limited extent through software. Groever et al. fabricated a doublet metalens with an NA of 0.44, focal length of 342.5 μm, and field of view of 50° for a wavelength of 532 nm [129]. TiO_2_ nanofins with the same length, width, and height are placed at different angles on a hexagonal lattice. The target phase profile was found using the PB phase. A resolution close to the diffraction limit was implemented, and there was no field curvature; therefore, the focal position of each field was the same, as shown in Fig. 7a–c. The larger the field of view, the larger is the number of aberrations that need to be compensated, thus making it difficult to realize a wide-angle metalens. Mikhail et al. used an aperture stop and metalens to achieve a 170° field of view and resolution close to the diffraction limit in the mid-infrared region [130]. The aberration was effectively compensated by reducing the overlapping part of the flux for each field in the metalens using the aperture stop. OpticStudio (Zemax, LLC) was used to obtain the initial layout and phase profile. A meta-atom satisfying the phase was found and placed appropriately. A PbTe meta-atom was fabricated on a 2-mm-thick CaF_2_ substrate by using electron-beam lithography, as shown in Fig. 7d–g. The focusing efficiency of the fabricated metalens was measured at 40% level for each field. The two studies mentioned above fabricated polarization-sensitive metalenses. Because the optical system is constructed using polarizers, the amount of incident light is reduced.

The focusing efficiency is one of the important parameters of a metalens. Reducing the incident light limits the extent to which the focusing efficiency can be increased. To overcome this limitation, some studies investigated polarization-insensitive metalenses [131–133]. Arbabi et al. demonstrated a polarization-insensitive metalens by constructing a silicon nanopost on a glass substrate, as shown in Fig. 7h [131]. It operates at a wavelength of 1550 nm, and the phase and transmission are adjusted by varying the diameter and lattice constant of the nanopost. The polarization-insensitive metalens was measured to have a very high focusing efficiency of 82%.

#### Chromatic aberration correction

**Fig. 8:**
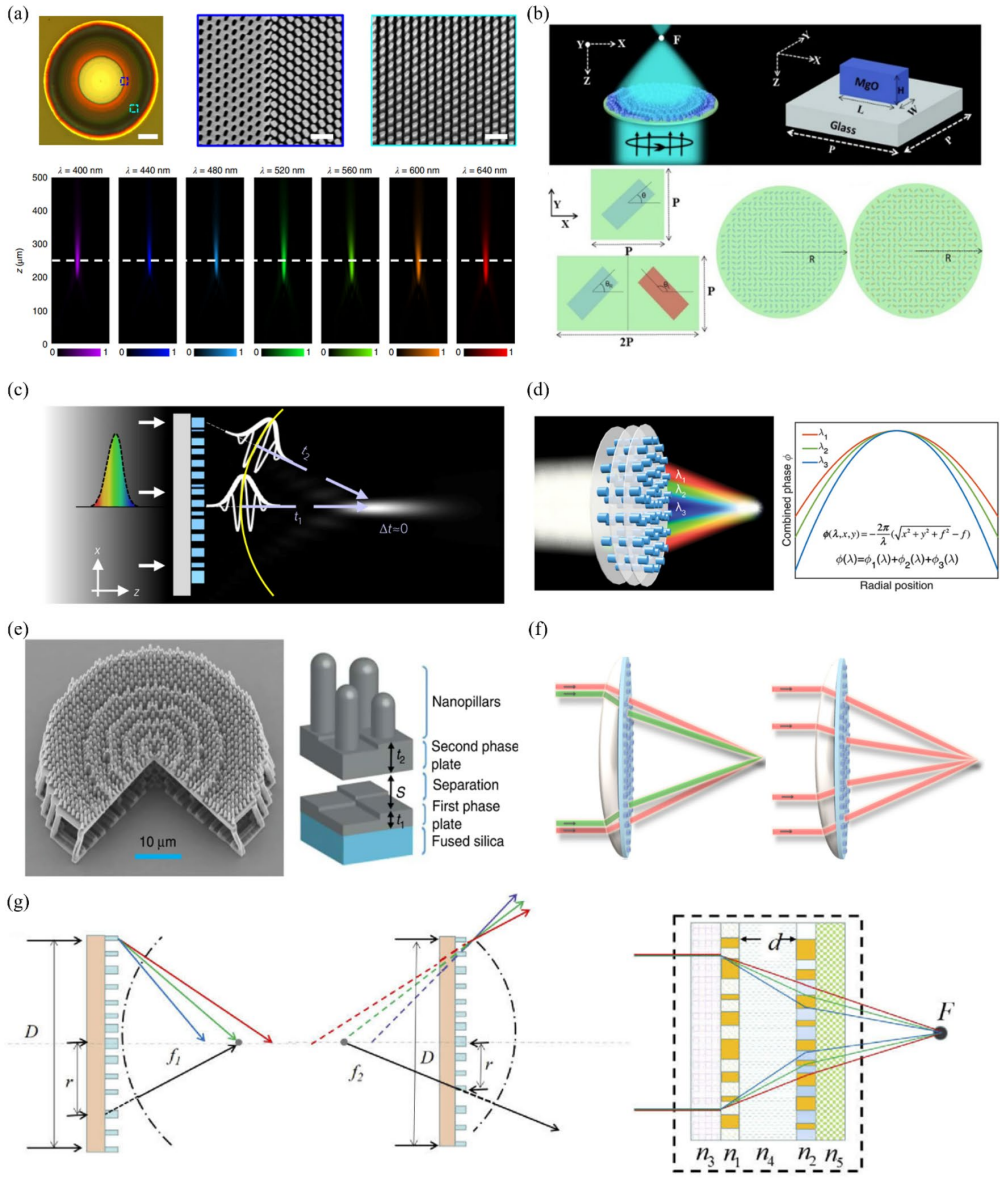
Chromatic aberration compensation lens: **a** SEM image of fabricated achromatic metalens with NA = 0.106. (top left) Entire metalens (scale bar: 10 μm), and (top middle and right) magnified view of inset of 1st image (scale bar: 500 nm). (bottom) Experimental light intensity profile according to incident light at each wavelength. White dashed lines indicate the focal plane. Reproduced with permission [99] (Copyright 2018, Springer Nature). **b** (top left) Achromatic metalens using CP light. (top right) MgO meta-atom with height H, width W, and length L on a uniform periodic P $\times$ P substrate. (bottom left) Unit cell of simple metalens and hybrid metalens. θ is the angle that satisfies the phase according to the geometric PB phase method. (bottom right) top view of simple and hybrid metalenses with radius *R*. Reproduced with permission [134] (Copyright 2021, Springer Nature). **c** Schematic of an achromatic metalens that satisfies Eq. (4). The metalens is designed such that wavepackets from different locations can reach the focus simultaneously. Yellow line indicates a spherical wavefront. Reproduced with permission [135] (Copyright 2018, Springer Nature). **d** Schematic of a multilayer dielectric metalens operating at multiple wavelengths. Each layer provides a required hyperbolic phase profile for each different wavelength. Reproduced with permission [136] (Copyright 2018, American Chemical Society). **e** (left) Schematic of hybrid metalens that combines a phase plate and metalens to compensate for chromatic aberration while improving focusing efficiency. (right) Unit cell of hybrid metalens. Reproduced with permission [137] (Copyright 2020, Springer Nature). **f** Hybrid achromatic metalens (scale: cm). (left) Chromatic aberration correction and (right) spherical aberration correction. Reproduced with permission [138] (Copyright 2021, Optica Publishing Group). **g** Schematic of a dual-layer achromatic metalens (DAML). Planoconvex metalens, planoconcave metalens, and cross-section of DAML. Reproduced with permission [139] (Copyright 2020, Optica Publishing Group)

Chromatic aberration is one of the major factors degrading the image quality, and therefore, it must be corrected. Chromatic aberration occurs because the refractive index of the medium varies depending on the wavelength. Recently, studies have actively investigated achromatic metalenses in the infrared [137, 140–143], visible [41, 99, 135, 138, 139, 144–147], and ultraviolet [134] regions.

The phase profile of an achromatic metalens is expressed as [99, 148]

11 $$\phi \left(r,\lambda \right)=-\left[\frac{2{\uppi }}{\lambda }\left(\sqrt{{r}^{2}+{f}^{2}}-f\right)\right]+{\phi }_{\text{s}\text{h}\text{i}\text{f}\text{t}}\left(\lambda \right)$$

12 $${\phi }_{\text{s}\text{h}\text{i}\text{f}\text{t}}\left(\lambda \right)=\frac{a}{\lambda }+b, a= \delta \frac{{\lambda }_{\text{m}\text{i}\text{n}}{\lambda }_{\text{m}\text{a}\text{x}}}{{\lambda }_{\text{m}\text{a}\text{x}-{\lambda }_{\text{m}\text{i}\text{n}}}} \text{a}\text{n}\text{d} b= -\delta \frac{{\lambda }_{\text{m}\text{i}\text{n}}}{{\lambda }_{\text{m}\text{a}\text{x}-}{\lambda }_{\text{m}\text{i}\text{n}} }$$

where $r$ is the radial coordinate on the metalens; *λ*, the wavelength; $f$, the focal length; and $\delta$, the largest additional phase shift. The ${\phi }_{\text{s}\text{h}\text{i}\text{f}\text{t}}\left(\lambda \right)$ term is introduced to optimize the phase compensation effect of an achromatic metalens. Equation 11 contains two terms related to the focusing and phase dispersion that are expressed as

13 $$\phi \left(r,\lambda \right)={\phi }_{f}\left(r,{\lambda }_{\text{m}\text{a}\text{x}}\right)+ {\phi }_{\text{d}\text{i}\text{s}\text{p}\text{e}\text{r}\text{s}\text{i}\text{o}\text{n}}\left(r,\lambda \right)$$

14 $${\phi }_{f}\left(r,{\lambda }_{\text{m}\text{a}\text{x}}\right)= -\left[\frac{2\pi }{{\lambda }_{\text{m}\text{a}\text{x}}}\left(\sqrt{{r}^{2}+{f}^{2}}-f\right)\right],$$

15 $${ \phi }_{\text{d}\text{i}\text{s}\text{p}\text{e}\text{r}\text{s}\text{i}\text{o}\text{n}}\left(r,\lambda \right)= -\left[2\pi \left(\sqrt{{r}^{2}+{f}^{2}}-f\right)\left(\frac{1}{\lambda }-\frac{1}{{\lambda }_{max}}\right)\right]+ {\phi }_{shift}\left(\lambda \right)$$

The PB phase is used to obtain ${\phi }_{f}\left(r,{\lambda }_{\text{m}\text{a}\text{x}}\right)$. The PB phase is generated when CP light is incident on a unit element with rotation. Therefore, if the PB phase is used, a phase independent of the wavelength can be obtained. In contrast, ${\phi }_{\text{d}\text{i}\text{s}\text{p}\text{e}\text{r}\text{s}\text{i}\text{o}\text{n}}(r,\lambda )$ is a function of the working wavelength, and it can be obtained by properly designing each unit element of the metalens. Because it is completely different from the PB phase used previously, it can be merged without mutual interference. By using the above method, a metalens with a broad band in the visible and ultraviolet region can be realized. Wang et al. demonstrated a broadband metalens with a focus efficiency of approximately 40% for an NA of 0.106, focal length of 235 μm, and wavelength region of 400–660 nm [99]. This lens integrated a PB phase and an integrated-resonant unit element (IRUE). IRUE is made of solid or inverse GaN-based structures on the subwavelength periodic hexagonal lattice, as shown in Fig. 8a. By carefully arranging and rotating the IRUEs on the substrate, the phase profile of the achromatic metalens can be satisfied. Ali et al. used the same principle to fabricate an achromatic MgO-based metalens with an NA of 0.8, focal spot of 182 nm, and wavelength region of 200–400 nm with a focus efficiency of 94%; it exhibited high transmission, ultrawide bandgap, and high refractive index in the ultraviolet region [134]. In addition, unit cells can be hybridized to control both LCP and RCP incident light, as shown in Fig. 8b.

Another strategy for correcting chromatic aberration is to simultaneously control the phase, group delay, and group delay disorder. The phase profile of a metalens is expressed as [135]

16 $$\phi \left(r,\omega \right)=-\frac{\omega }{c}\left(\sqrt{{r}^{2}+{f}^{2}}-f\right)$$

where *ω*, *c*, $r$, and $f$ are the angular frequency, light speed, radial coordinate on the metalens, and focal length, respectively.

The Taylor series expansion of this equation around the design frequency ${\omega }_{\text{d}}$ is expressed as

17 $$\phi \left(r,\omega \right)= \phi \left(r,{\omega }_{\text{d}}\right)+ {\left.\frac{\partial \phi \left(r,\omega \right)}{\partial \omega }\right|}_{\omega ={\omega }_{\text{d}}}\left(\omega -{\omega }_{\text{d}}\right)+ {\left.\frac{{\partial }^{2}\phi \left(r,\omega \right)}{2{\partial }^{2}\omega }\right|}_{\omega ={\omega }_{\text{d}}}{\left(\omega -{\omega }_{\text{d}}\right)}^{2}+\cdots$$

where $\phi$ is the target phase profile; $r$, the radial coordinate on the metalens; and ${\omega }_{\text{d}}$, the angular frequency of the designed frequency.

The first term on the right-hand side represents the phase profile of the metalens. The second term, namely, the group delay, compensates for the difference in the arrival time of wave packets at the focal point. The last term, namely, the group delay dispersion, guarantees identical outgoing wavepackets. In general, these have an order of $fs$and ${fs}^{2}$ in the visible area. The group delay and group delay dispersion are the main factors causing chromatic aberration. Therefore, to demonstrate a metalens in a broad band, these two terms must be appropriately compensated, as shown in Fig. 8c.

Chen et al. fabricated a broadband achromatic metalens. They achieved near diffraction limit performance by using a single metalens with a thickness of the order of the wavelength for an NA of 0.2 and wavelengths region of 470–670 nm. One or more TiO_2_ nanofins are present in the metalens element. The nanofins were fixed at a height of h = 600 nm and distance p = 400 nm, and their length, width, and rotation angle were designed differently. The efficiency was approximately 20% at a wavelength of 500 nm; the aim is to improve the efficiency further. The group delay has the same relationship as the NA and diameter [135]:

18 $$\left|\frac{\partial \phi }{\partial {\upomega }}\right|= \frac{R \times NA}{2c}$$

Therefore, some limitations are faced in implementing a large-sized achromatic metalens. To overcome these limitations, studies are investigating multilayer and hybrid metalenses [136–139, 144–146].

Zhou et al. developed a multilayer dielectric metalens with NA of 0.42 that operates at multiple wavelengths in the infrared region (i.e., 1100 nm, 1400 nm, and 1680 nm) [136]. They achieved a focal efficiency of 1180 nm (48% design, 38% measurement) and 1680 nm (56% design, 52% measurement). A two-layer metalens was constructed with Si nanoposts, and the desired phase was found by combining the radii of the cylindrical meta-atoms of each layer, as shown in Fig. 8d.

Balli et al. fabricated a 300-µm-diameter hybrid achromatic metalens (HAML) that operated in the infrared region between 1000 and 1700 nm [137]. By using Nanoscribe Photonic Professional GT and IP-Diphotoresist, they constructed a substrate–phase plate–airgap–phase plate–nanopillar form to realize a metalens with high NA and large diameter, as shown in Fig. 8e. The achieved an average and maximum focal efficiency of more than 60% and 80%, respectively.

Sawant et al. demonstrated an achromatic hybrid centimeter-scale metalens in combination with a conventional lens, as shown in Fig. 8f [138]. They effectively compensated the chromatic aberration by using the property of negative dispersion of diffractive optical elements. The metalens was manufactured using a negative electron-beam resist (ma-N 2410). Because an exposed resist was used, the lens could be produced faster than with electron-beam lithography. Although the focal efficiency was low at 8.5%, it showed the possibility of implementing an achromatic hybrid metal at centimeter scale, as shown in Fig. 8f.

Li et al. demonstrated a DAML design with an effective Abbe number [139]. Chromatic aberration was corrected by combining a planoconvex metalens (PVML) and a planoconcave metalens (PCML) with the lens maker equation. The PVML contains GaN embedded in an Al_2_O_3_ substrate (height: 1400 nm, period: 195 nm), and the PCML contains a GaN cylinder on the substrate (height: 900 nm, period: 195 nm). The use of a cylindrical meta-atom enables the implementation of a polarization-independent achromatic metalens. In the visible region, a focal length difference of 2 μm for each wavelength and focus efficiencies of over 50% were achieved, as shown in Fig. 8g.

## Applications

### Imaging system

High-performance lenses are needed to obtain high-quality images. A high-performance lens can be realized by using several lenses or aspherical lenses in a traditional method. The use of multiple lenses can effectively compensate for aberrations; however, it has the disadvantage of increasing the weight and size. This is particularly an issue for application to mobile phone cameras, for which the weight and size must be reduced. Therefore, the number of lenses can be increased only to a limited extent. For aberration compensation, aspherical lenses are advantageous compared with spherical lenses; however, their manufacturing process and high cost remain problems. Further, some limitations are faced in their miniaturization. Therefore, studies are actively investigating means of obtaining high-quality images with a metalens as it is very compact compared with conventional lenses.

**Fig. 9:**
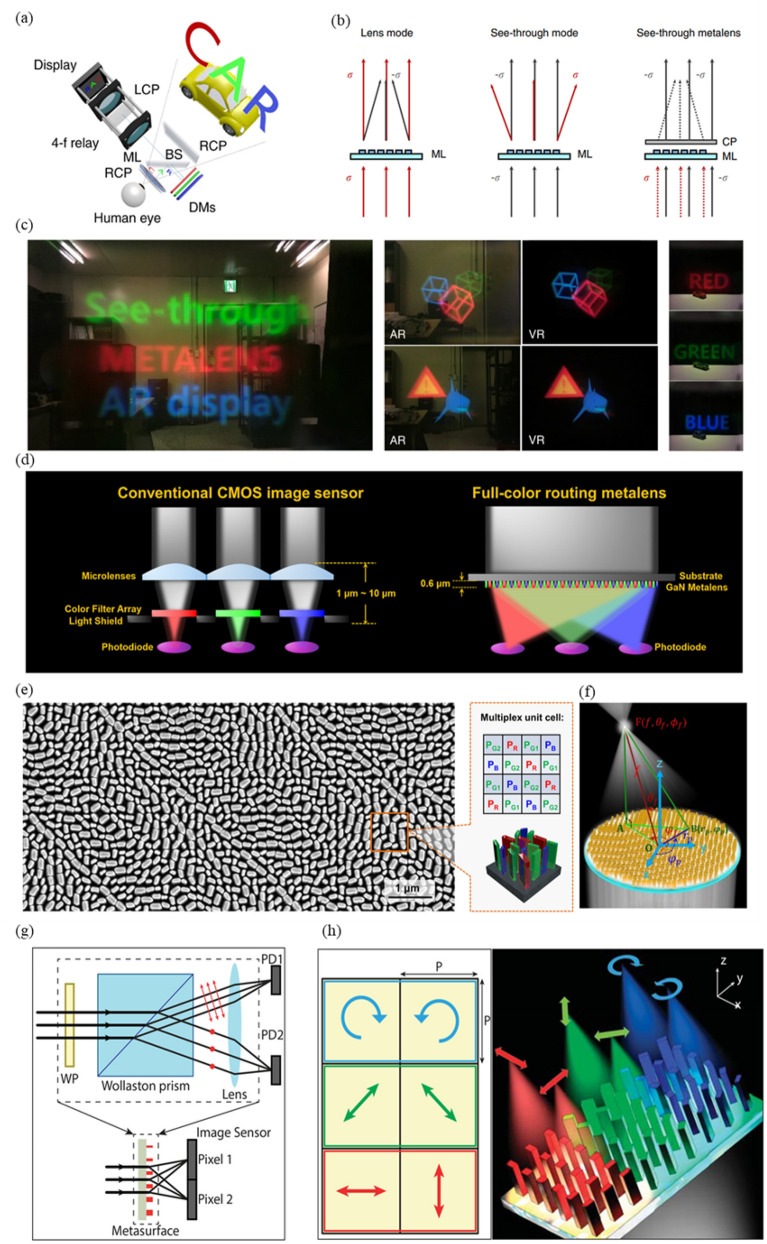
Imaging system using metalens: **a** Schematic of see-through near-eye display using metalens (ML), dichroic mirrors (DMs), beam splitter (BS), LCP, and RCP. **b** Schematic of operation principle of a metalens according to the incident direction of CP light and when combined with a circular polarizer. **c** Augmented reality (AR) images that combine a real object and virtual information and virtual reality (VR) images that only show virtual information with a fabricated achromatic metalens with NA = 0.106. **a**-**c** are reproduced with permission [128] (Copyright 2018, Springer Nature). **d** (left) Conventional CMOS image sensor (CIS) with microlenses and color filters and (right) CIS with full-color routing metalens. **e** SEM image of full-color routing metalens and a schematic of a multiplex unit cell. **f** Schematic of metalens causing the convergence of incident light at an arbitrary location F. O: center of metalens, B: arbitrary position on metalens surface, A: vertical projection point from focal point F onto metalens surface. **d**-**f** are reproduced with permission [100] (Copyright 2017, American Chemical Society). **g** (top) Schematic of conventional setup. WP : waveplate, PD : photodetector. (bottom) schematic of using metasurface. **h** Schematic diagram of a supercell with different polarization focus at different points. **g** and **h** are reproduced with permission [149] (Copyright 2018, American Chemical Society)

#### Near-eye display system

Recently, with the development of AR and VR technologies, high-performance and miniaturized near-eye displays have attracted increasing attention [150–153]. Both AR and VR should have a small display size, and the size of the virtual image created through the optical system should be large. To satisfy these requirements, a reflective optical system, Fresnel lenses, diffractive optical elements, etc. can be used. However, using conventional lenses has limitations in reducing the size [154, 155]. Therefore, compact AR and VR were implemented using a metalens, as shown in Fig. 9a–3c [128].

AR is emerging as a next-generation display technology that combines the real world and virtual information [150]. An important factor in AR is to combine these two without producing a sense of difference and to have a small size so that users do not feel uncomfortable wearing it.

Lee et al. implemented a see-through AR near-eye display by using a see-through metalens [128]. This display had an ultrawide FOV, full-color imaging, high resolution, and sufficiently large eyebox. Figure 9a shows how objects in the real world and virtual information are combined, and Fig. 9b shows how the see-through near-eye display works. When light with circular polarization of $\sigma$is incident, the metalens acts as a convex lens for cross-polarized transmission and transmits it for co-polarized transmission. Conversely, when light with -$\sigma$ circular polarization is incident, it works as a concave lens for cross-polarized transmission and transmits it for copolarized transmission. Therefore, when any CP light is incident, it has a total of four transmitted light components. If the $\sigma$ direction is blocked using a circular polarizer, only two elements remain. Light incident in the $\sigma$ direction is converged, and light incident in the -*σ* direction is transmitted. Two pieces of information can be combined by making the virtual information in the $\sigma$ direction and the real-world information in the -$\sigma$ direction incident on the metalens. Because the see-through metalens has different focal lengths depending on the wavelength, full-color imaging cannot be performed on the same focal plane, as shown in Fig. 9c; however, it could be performed by correcting the color aberration by using a dichroic mirror. First, a polymer stamp was fabricated with polyurethane-acrylate (PUA) MINS-311 RM (Minuta Technology Co.) imprint resin and silicon master fabricated by electron-beam lithography, and Au, Cr and SiO2 were deposited on the polymer stamp. Next, the poly-crystalline silicon (Poly-Si) was deposited on the quartz wafer substrate using low-pressure chemical vapor deposition (CVD). Adhesive and polymer stamp were placed on the substrate and transferred using a roll-to-plate system (Eastern Engineering). The transferred Cr is removed after being used as a hard mask for etching Poly-Si. Finally, any remaining residues were removed through an etching process, completing the metasurface device.

#### Pixel-level full-color router

Most imaging systems use CISs. A CIS detects the intensity of light with a photodiode; however, it cannot distinguish colors. Therefore, color filters and microlenses are used to distinguish colors. The former disperses light with different wavelengths, and the latter increases the light collection efficiency. The distance from the vertex of the microlens to the rear of the color filter array is 1–10 μm. Therefore, the CIS is bulky. A full-color routing metalens has been announced to improve this issue, as shown in Fig. 9d [100]. This device has high efficiency, narrow bandwidth, and different focusing positions depending on the wavelength. Figure 9e shows the structure of the metalens and multiplex unit cell. Because most CISs use a Bayer filter, the full-color router’s multiplex unit cells were similarly arranged in RGBG, as shown in the right-hand side of Fig. 9e. A metalens was fabricated with a square size of 50 μm × 50 μm; focal length of 110 μm; polar angle *θ*_*f*_ of 8°; and azimuthal angles ${\phi }_{f, \text{R}}$ = 45°, ${\phi }_{f, \text{G}1}$ = 135°, ${\phi }_{f, \text{B}}$ = 225 °C, and ${\phi }_{f, \text{G}2}$ = 315°, as shown in Fig. 9f. The efficiency was measured by using lasers of each wavelength in a full-color router; the measured efficiencies for R (633 nm), G1 (532 nm), B (430 nm), and G2 (532 nm) were 15.9%, 37.86%, 38.33%, and 27.56%, respectively. If a full-color router is used, it can be implemented compactly by integrating color filters and microlens arrays. In addition, because the color filter is not used, no light is absorbed before being incident on the photodiode (e.g., blue and green absorption when passing through the red filter). Therefore, the efficiency of light incident on the photodiode can be increased. The manufacturing method is as follows. A 600 nm undoped GaN layer was grown on a c-plane 430 μm thick sapphire substrate using metal-organic chemical vapor deposition (MOCVD). Then, a 400 nm SiO2 hard mask was deposited using plasma-enhanced chemical vapor deposition (PECVD), and a ZEP520A layer was spin-coated to a thickness of 100 nm and baked at 180 °C for 2 min. To reduce positional errors, a highly conductive organic polymer layer, Espacer, was stacked. The structural properties of the metasurface are created by the ZEP-N50 e-beam exposure process. Then, a 40 nm Cr layer is coated as an etching hard mask and followed by lift-off process using ZDMAC. The patterns are transferred to the 400 nm thick SiO2 layer by reactive ion etching (RIE). Finally, the SiO2 hard mask layer is etched by inductively coupled-plasma reactive ion etching (ICP-RIE) using BCl3/Cl2 chemistry and removed with buffered oxide etch.

#### Division of focal plane polarization camera

As mentioned in the multifunctionality section, metasurfaces are easy to implement independent functions according to polarization, and thus can be applied to polarization cameras to realize polarimetric imaging. Arbabi et al. used a metasurface to demostrate a division of focal plane polarization camera (DoFP-Pc) [149]. In Fig. 9g, the concepts of DoFP-Pc with the conventional method and metasurface are introduced. In general, DoFP-Pc is composed of two optical elements, one for polarization control and the other for phase control. Despite its bulky nature, it can be rendered more compact by integrating both polarization and phase control into a single metasurface. The metasurface is composed of rectangular cross-sections with a height of 650 nm a-Si nanoposts, as shown in Fig. 9h. By varying the width, length, and angle of each nanopost, full and independent 2π phase control was achieved. The DoFP-Pc is designed to split light for three polarizations and have a different focus. Therefore, it achieved 60 ~ 65% efficiency, which is higher than the theoretical efficiency limit of 50% of the polarization filtering method. In the method of manufacturing the metasurface, 650 nm a-Si was deposited on a 500 μm fused silica substrate and patterned 300 nm-thick ZEP-520 A using electron-beam lithography, and 70 nm aluminum oxide was used as a hard mask to invert the pattern.

### Spectrometer

**Fig. 10:**
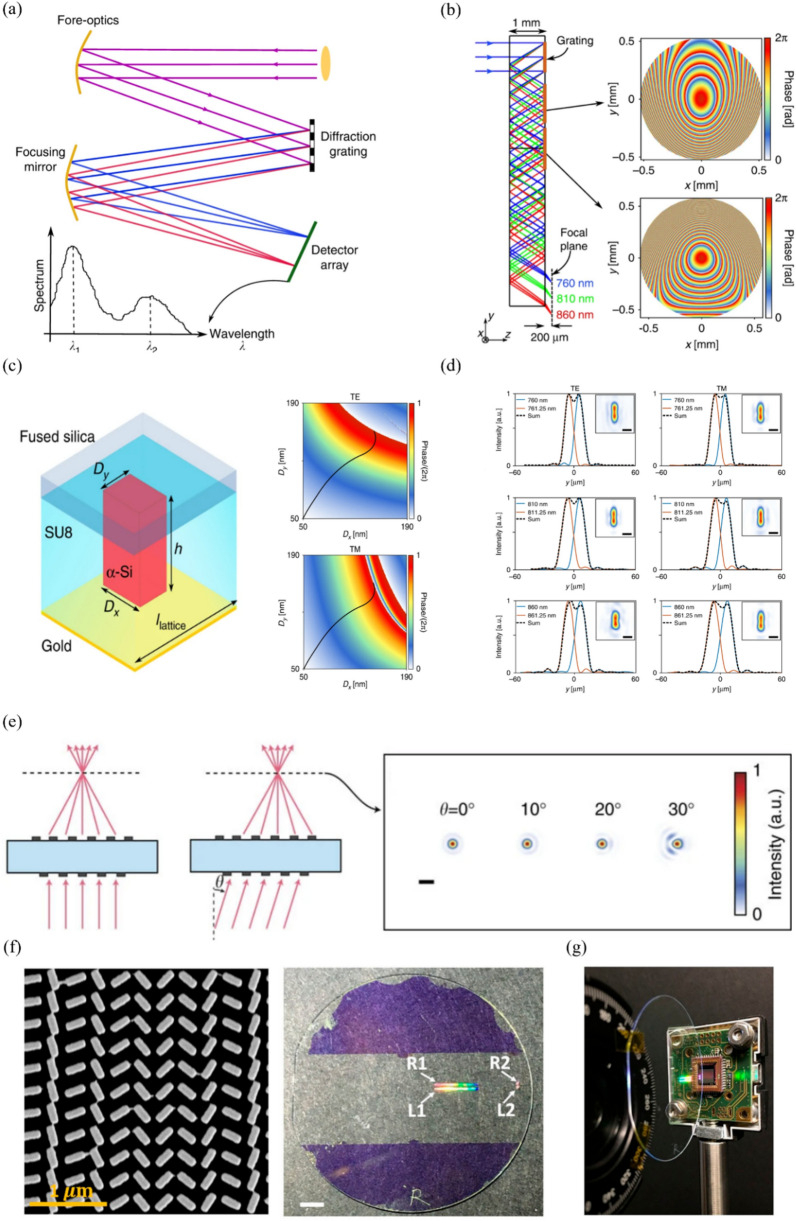
Spectrometer using metasurface: **a** Schematic of a conventional spectrometer. **b** Schematic of compact spectrometer using metasurface, and phase profile of metasurface compensating for monochromatic aberration. **c** (left) Structure of unit cell, and (top right and bottom) simulated phase according to TE and TM polarization, where the black curve represents D_x_-D_y_ with the same phase for TE and TM polarization. **d** Intensity distribution when light with wavelength difference of 1.25 nm is incident with TE and TM polarization. **a**-**d** are reproduced with permission [156] (Copyright 2018, Springer Nature). **e** Schematic of a doublet metalens for on-axis and off-axis incidence, where the spot size is small owing to monochromatic aberration compensation (scale bar: 2 μm). Reproduced with permission [157] (Copyright 2016, Springer Nature). **f** (left) SEM image of fabricated metalens, and schematic of fabricated device with four separate metalenses. (right) R and L refer to the helicity of light focused by each metalens, and 1 and 2 indicate the parameters used for the lens design (scale bar: 5 mm). **g** Compact spectrometer that combines a metalens and a CMOS camera. **f** and **g** are reproduced with permission [158] (Copyright 2017, AIP Publishing)

A spectrometer is used in various fields of study. Accordingly, spectrometers with different size, resolution, and operation bandwidth are required. A conventional spectrometer consists of a prism or diffraction grating that refracts light of various wavelengths at different angles and focusing elements that focus light incident at different angles to different points. Figure 10a shows a schematic diagram of a conventional spectrometer having a large overall size with a space for arranging optical elements and a space for propagating light. The compactness of a conventional spectrometer is limited. Therefore, the use of a metasurface has been explored to realize a compact spectrometer [156]. Faraji-Dana et al. demonstrated a compact spectrometer with a thickness of 1 mm and volume of 7 mm^3^ [156]. In the 760–860 nm near-infrared region, they produced more than 80 spectral points with a resolution of ~ 1.2 nm. Figure 10b shows the ray tracing simulations of the designed spectrometer. Three metasurfaces are located on one side of the 1-mm fused silica substrate. Each meta-atom consists of an a-Si nanopost with a rectangular cross-section, with a 2-µm-thick SU-8 layer capped and supported by a gold mirror, as shown in Fig. 10c. The uppermost metasurface in Fig. 10b is a blazed grating with a 1-µm period. This metasurface disperses parallel incident light of different wavelengths at different angles. The second and third metasurfaces focus incident light of different angles for each wavelength at different positions. In addition, these two metasurfaces compensate for monochromatic aberrations like a doublet metalens to achieve high resolution, as shown in Fig. 10e [157]. Figure 10d shows the intensity measurement result when two wavelengths with a wavelength difference of 1.25 nm are incident in TE and TM modes. Zhu et al. fabricated a compact spectrometer in the visible light region by using a CMOS camera and metalenses [158]. Specifically, they fabricated a metalens with NA of 0.1 for a high spectral resolution and a metalens with NA of 0.022 for a wide spectral range. The two metalenses were selectively focused according to the direction of the incident circular polarization because the PB phase was used. These metalenses were placed suitably on the substrate such that their focal spots did not overlap, as shown in Fig. 10f. Finally, the metalenses were combined with a CMOS camera (Thorlabs DCC1545M) to realize a compact spectrometer, as shown in Fig. 10g. It was fabricated using conventional micro- and nano-fabrication technology. First, 395 nm a-Si was deposited on a fused silica substrate with a thickness of 1 mm. Next, the metasurface was patterned using electron beam lithography, and the pattern was inverted through lift-off and dry etching processes. Finally, it was covered with a 2 μm-thick SU-8 layer, and a gold layer for reflection was deposited on both sides.

## Conclusion and outlook

This paper discussed the principle, functionality, and application of metalenses. The principles were discussed in terms of the materials used, phase modulation, and design. The wavelength range of the metalens differs depending on its application. Materials suitable to a wavelength range must be selected in terms of the real and imaginary parts of the complex refractive index. Phase modulation methods are classified into the resonant phase, propagation phase, and geometric phase. Forward design is a traditional design method, and inverse design can be used to realize more advanced and large-scale metalenses because it offers a larger number of degrees of freedom compared to forward design. Various functionalities of metalenses, including multifunctionality, tunability, high NA, integration, and aberration correction were discussed. Metalenses can integrate the functionalities of light’s polarization, intensity, and OAM, which cannot be achieved by conventional lenses. In terms of multifunctionality and tunability, metalenses introduce new capabilities that are not possible with traditional lenses. Moreover, metalenses can achieve high NA through rapid phase modulation. The Integration section introduces new functionalities that combine metalenses with existing optical components, including high-NA metafiber systems, achromatic metafibers, waveguide-integrated one-dimensional resonance-phase-based metalenses, and waveguide-integrated two-dimensional metalenses. These technologies have the potential to enhance the performance and functionality of optical systems by integrating multiple functionalities into a single element. In the Aberration Correction section, we introduce methods for correcting monochromatic and chromatic aberrations using metalenses. Moreover, we review the studies that have implemented designs such as doublet metalenses, fisheye metalenses, and hybrid metalenses to further improve the correction of aberrations. Finally, studies that used these functionalities for various applications were introduced.

Recently, imaging systems are finding increasing application in wearable devices, self-driving cars, AR, VR, and mobile phones. Most such applications require high performance and a compact size. Currently, these requirements are being satisfied with conventional or diffractive lenses. However, such lenses have a bulky size, and their miniaturization limits their performance. A metalens can overcome these limitations because it affords both high performance and a compact size. Of course, there are limitations such as operating bandwidth, focusing efficiency, large-scale metalens design and manufacturing method, and high price, but studies using metalens are actively being conducted to overcome these limitations. Thus far, studies have actively investigated the functionalities of metalenses, and now, they are actively investigating applications of metalenses [57, 159–172]. We expect that metalenses with ultrahigh performance and compact sizes will replace existing optics in the future.

## Data Availability

Not applicable.
